# Identification of tRF-29-79MP9P9NH525 as a biomarker and tumor suppressor of gastric cancer via regulating KIF14/AKT pathway

**DOI:** 10.1038/s41420-025-02514-9

**Published:** 2025-05-15

**Authors:** Jiaxin Ge, Ji Dai, Haoqiang Ji, Jie Guo, Xiaoban Shen, Desen Sun, Qiang Chen, Pan Chen, Guoliang Ye, Junming Guo, Shuangshuang Zhang

**Affiliations:** 1 Department of Gastroenterology, The First Affiliated Hospital of Ningbo University, 315020 Ningbo, China; 2 Department of Biochemistry and Molecular Biology, School of Basic Medical Sciences, Health Science Center, Ningbo University, 315211 Ningbo, China; 3 The First Department of General Surgery, Ningbo Zhenhai People’s Hospital, 315202 Ningbo, China; 4 School of Materials Science & Chemical Engineering, Ningbo University, 315211 Ningbo, China; 5 Ningbo Institute of Innovation for Combined Medicine and Engineering, The Affiliated Lihuili Hospital of Ningbo University, 315100 Ningbo, China

**Keywords:** Cell biology, Cancer

## Abstract

Gastric cancer (GC) is one of the most common malignancies with a poor prognosis. The development of novel biomarkers is of utmost importance to screen patients with GC. Molecular mechanism study of GC may provide a research basis for the development of targeted drugs. We identified tRF-29-79MP9P9NH525 (tRF-29) as a GC-associated tRNA-derived fragment (tRF). The specific hair-pin structure reverse primer and amplification primers were first designed and then applied for tRF-29 quantification. Receiver operator characteristic curve, Kaplan–Meier survival curve, and multivariate Cox analysis were applied to analyze the diagnostic and prognostic values of tRF-29 in GC. Ethynyl-2′-deoxyuridine, cell cloning, Transwell assay, and flow cytometry were used to detect the effects of tRF-29 on proliferation, migration, and cell cycle distribution of GC cells. Xenograft tumor formation in NOD-SCID mice was applied in determining tRF-29′s effects on tumor growth. Fluorescence in situ hybridization, dual luciferase reporter assay, Western blot, immunohistochemistry, and RNA-binding protein immunoprecipitation were conducted to explore the molecular mechanism underlying tRF-29 regulating GC development. It was found that tissue tRF-29 showed effective diagnostic efficiency in GC and could discriminate different gastric mucosa. Besides, plasma tRF-29 improved GC diagnostic values of common tumor markers and had prognostic values in GC. tRF-29 was found to suppress proliferation and cell cycle progression. tRF-29 inhibited the growth of xenograft tumors. Mechanically, tRF-29 exerted Kinesin family member 14 (KIF14) mRNA destabilization by combining with argonaute 2 (Ago2) and regulated AKT/P27 pathway. In conclusion, tRF-29 inhibited GC progression by combining with Ago2 and regulated AKT/P27 pathway by silencing KIF14 expression.

**Figure:**
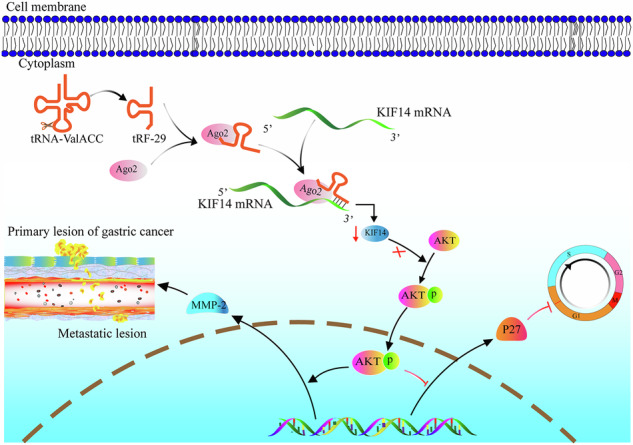
In normal cells, tRF-29, derived from tRNA-ValACC, targets the 3’UTR region of KIF14 mRNA by forming RNA silencing complex with Ago2. Reduced KIF14 results in less phospholation of AKT. Subsequently, the expression of P27 is increased, while the expression of MMP-2 is decreased. Finally, the cell cycle is arrested, and the cell proliferation is suppressed, as well as the metastasis is inhibited. In gastric cancer cells, due to the downregulated of tRF-29, the expression of KIF14 is increased, thus the cell proliferation and metastasis are promoted via AKT pathway.

In normal cells, tRF-29, derived from tRNA-ValACC, targets the 3’UTR region of KIF14 mRNA by forming RNA silencing complex with Ago2. Reduced KIF14 results in less phospholation of AKT. Subsequently, the expression of P27 is increased, while the expression of MMP-2 is decreased. Finally, the cell cycle is arrested, and the cell proliferation is suppressed, as well as the metastasis is inhibited. In gastric cancer cells, due to the downregulated of tRF-29, the expression of KIF14 is increased, thus the cell proliferation and metastasis are promoted via AKT pathway.

## Introduction

Gastric cancer (GC) is the most common type of gastrointestinal malignancy and accounts for one in every 13 deaths worldwide [1]. Attributed to a deficiency of particular clinical characteristics or signs in the early stage, most patients miss the best diagnostic and treatment time, resulting in the progression of GC to a late stage. However, no specific biomarker is available for GC screening or for prognosis evaluation [2]. Neoadjuvant therapy is the first choice in GC treatment [3]. The molecular understanding of GC has led to renewed optimism that targeted agents can improve survival rates and reduce chemotherapy toxicity [4]. For example, human epidermal growth factor-2 (HER-2) positive GC patients may benefit from the application of transtuzumab [5]. Ramucirumab, an inhibitor of the receptor for vascular endothelial growth factor (VEGF) was shown to improve prognosis of advanced GC patients [6]. The development of GC is a progressive process, and its pathogenesis is multi-factorial and involves complex biological network regulation. As a result, expounding the underlying mechanism of GC occurrence may help to explore novel targeted molecular drugs.

Non-coding RNAs (ncRNAs) have been recognized as crucial participators in diverse aspects of biological processes. tRNAs are conventional ncRNAs that have definite roles in protein translation [7]. Recently, the advancement of high-throughput sequence techniques has brought about the discovery of novel types of small RNAs, including tRNA-derived fragments (tRFs). Contrary to previous conceptions that tRFs are arbitrary debris of tRNAs, they were identified as specific ncRNA that exhibit tissue-specific expression and have distinct functions in diseases [8, 9]. Based on the disparate cleavage sites on tRNA, tRFs can be classified into 3′U tRFs, i-tRFs, 5′-tRFs, and 3′-tRFs, etc. [10]. Accumulating evidence demonstrates that functionally diverse tRFs contribute to the occurrence and development of tumors via regulating genes expression [11–13]. In the previous study, we found that tRF-33-P4R8YP9LON4VDP exerts signal transducer and activator of transcription 3 (STAT3) silencing in argonaute 2 (Ago2) dependent manner [14]. Moreover, evolving evidences have confirmed the aberrant expression profiles of tRFs in different types of human tissues or body fluids [15, 16], which suggested the potential of tRFs in monitoring tumors [17].

The expression of tRFs was identified aberrantly in the plasma of GC patients comparing with that in healthy controls in our previous study [18]. In this study, we identified tRF-29-79MP9P9NH525 (tRF-29), a novel tRNA^ValACC^-derived tRF. We found that tissue tRF-29 showed effective diagnostic efficiency in GC and could discriminate different gastric mucosa. Besides, plasma tRF-29 showed diagnostic and prognostic values in GC. The diagnostic efficiency was significantly improved when tRF-29 was combined with carcinoma embryonic antigen (CEA), α-fetoprotein (AFP), carbohydrate antigen 125 (CA125), or CA19-9. In vitro experiments showed that tRF-29 suppressed GC cell proliferation. Its inhibitory effects were further determined in the xenograft tumor formation. Furthermore, we revealed that tRF-29 destabilized Kinesin family member 14 (KIF14) mRNA and regulated the AKT pathway by interacting with Ago2.

## Results

### Establishment of tRF-29-79MP9P9NH525 detection system

**Fig. 1:**
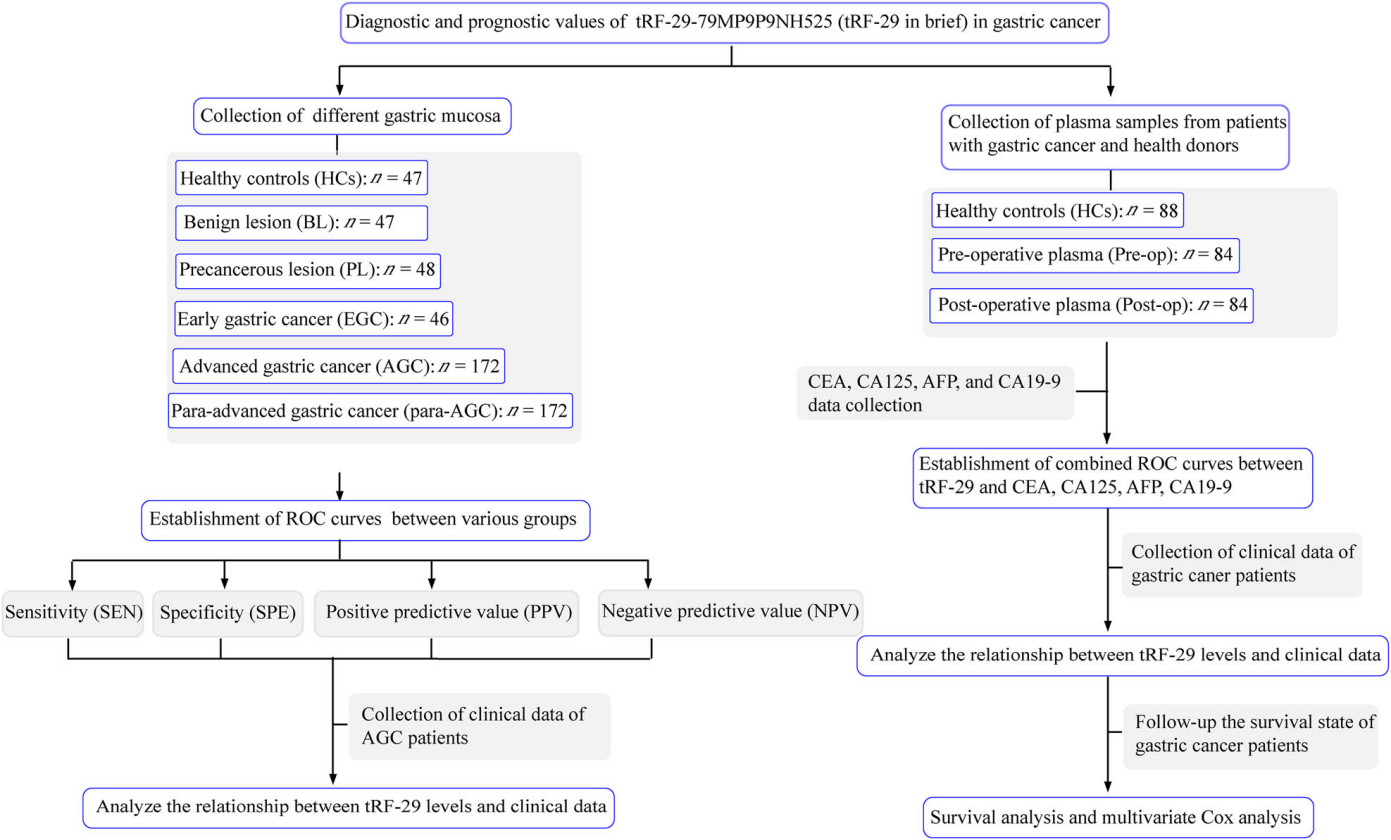
Flow chart of the study design. ROC receiver operator characteristic.

This study examined the clinical significance of tRF-29 in diagnosis and prognosis assessment of GC (Fig. 1). tRF-29 is a type of 5′-tRF with the sequence of 5′-GCUUCUGUAGUGUAGUGGUUAUCACGUUCGC-3′ (Supplementary Fig. 1A). Since its size is too short to detect by conventional qRT-PCR, loop sequence and a pair of complementary sequences of 5′-GCAGAGACGGGGTA-3′ and 5′-TACCCCGTCTCTGC-3′ were designed to form hairpin-loop primer for tRF-29 transcription. During the process of transcription, 3′-sticky with the sequence of 5′-GAACGT-3′ linked to the tRF-29 3′-termini to extend the single-stranded template of cDNA (Supplementary Fig. 1B). Then the amplimers were designed for tRF-29 quantification (Supplementary Table 1). The sequencing data of the qRT-PCR products validated the accuracy of the primers (Supplementary Fig. 1C). Agarose gel electrophoresis was conducted on the tRF-29 qRT-PCR product, revealing the presence of a single band (Supplementary Fig. 1D).

### tRF-29-79MP9P9NH525 was reduced in both gastric cancer tissues and patients’ plasma

**Fig. 2:**
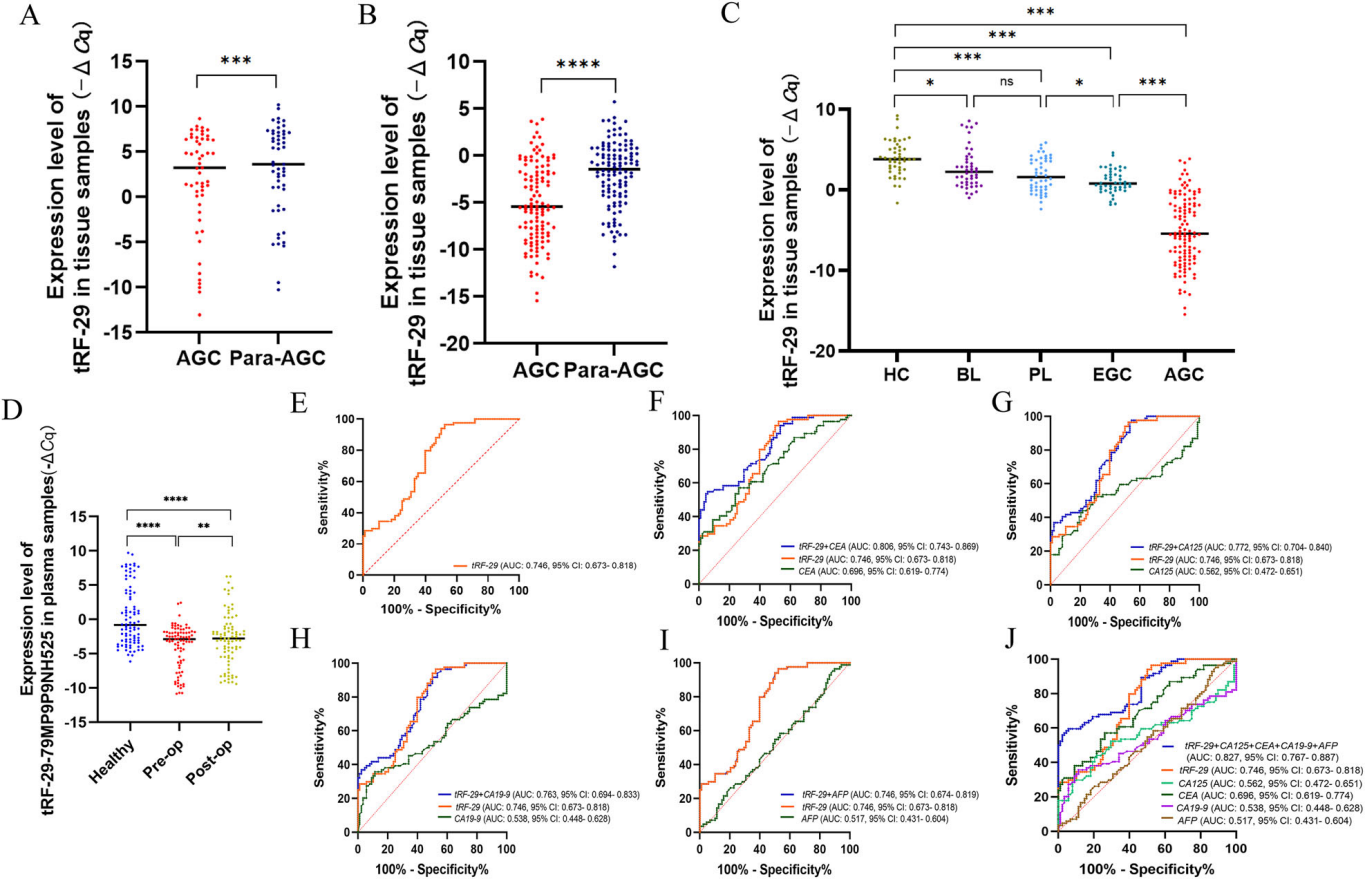
The abundance and clinical values of tRF-29-79MP9P9NH525 (tRF-29) in various samples. **A** tRF-29 was downregulated in advanced gastric cancer (AGC) tissues than in adjacent normal tissues. Training set, *n* = 53. **B** tRF-29 also showed a reduced level in enlarged AGC group. Validation set, *n* = 119. **C** tRF-29 level showed a gradient change from HCs group to AGC group. Healthy controls (HCs): *n* = 47, Benign lesion (BL): *n* = 47, Precancerous lesion (PL): *n* = 48, Early gastric cancer (EGC): *n* = 46, Advanced gastric cancer (AGC): *n* = 119. **D** tRF-29 abundance was observed in plasma samples from pre-operative (Pre-op), post-operative (Post-op) gastric cancer patients and healthy controls. Healthy: *n* = 88; Pre-op: *n* = 84; Post-op: *n* = 84. **E** Receiver operator characteristic (ROC) curve of tRF-29. **F**–**I** The joint ROC curves of tRF-29 and carcinoembryonic antigen (CEA), carbohydrate antigen 125 (CA125), CA19-9, and α-fetoprotein (AFP), respectively. **J** The joint ROC analysis of tRF-29, CEA, CA125, CA19-9, and AFP. *P < 0.05, **P < 0.01, ***P < 0.001, ****P < 0.0001; ns no significance.

To study the possible diagnostic and prognostic evaluation values of tRF-29 in GC patients, we examined tRF-29 levels in 53 pairs of AGC samples and matching adjacent non-tumor tissues as training set. tRF-29 was found significantly downregulated in the AGC samples (Fig. 2A). Then, we extended the tissue samples to 119 pairs of AGC samples and adjacent non-tumor tissues as a validation set. tRF-29 also showed lower levels in the AGC group than those of controls (Fig. 2B). Given that the progression of gastric cancer is a gradual process, we pondered the relationship between tRF-29 levels and GC occurrence. As a result, we assessed the tRF-29 abundance in tissue specimens from patients with BL, PL, EGC, or AGC, as well as HCs. Intriguingly, tRF-29 showed a gradient change from HC to AGC group (Fig. 2C). For details, tRF-29 showed a significant decrease of tRF-29 expression in AGC and EGC tissues compared to HCs, BLs and PLs (Fig. 2C). Meanwhile, the expression of tRF-29 in PLs was found to be lower in comparison to HCs or BL (Fig. 2C). The results suggested the potential of tRF-29 for differentiating different gastric pathologic stages. The gradient change of tissue tRF-29 levels indicated its potential to be biomarker in distinguishing patients with GC from normal controls. We wondered whether tRF-29 could be detected at a differential level in plasma from patients with GC and healthy donors. Then tRF-29 levels in plasma from patients with GC 1 day before and 9 days after surgical resection were detected. As indicated in Fig. 2D, the level of tRF-29 in preoperative and postoperative plasma was significantly lower than that in healthy controls, while its level in postoperative plasma was slightly higher than that in pre-operative plasma.

### The clinical significance of tRF-29-79MP9P9NH525 in GC

To comprehensively grasp the clinical relevance of tissue tRF-29 levels in monitoring various gastric mucosa conditions. We constructed the ROC curves for all tissue groups to analyze the distinguishing ability of tRF-29 for different gastric mucosa (Supplementary Fig. 2). The area under ROC curve (AUC) was 0.889, 0.926, and 0.970 in differentiating AGC from PL, BL, and HCs, respectively. The sensitivity and specificity of tRF-29 distinguishing AGC from PL was 68.1% and 95.8%, distinguishing AGC from BL was 83.2% and 91.5%, distinguishing AGC from HCs was 93.3% and 93.6%, respectively. Furthermore, tRF-29 also performed well at differentiating EGCs from BL and HCs, with the AUCs being 0.706 and 0.877, and the sensitivity to be 63.0% and 93.5%, respectively. We also analyzed the application of tRF-29 in differentiating PL, BL, and HCs. tRF-29 showed an AUC of 0.756 in identifying PL from HCs. While there′s no significant values in distinguishing PL from BL, or BL from HCs.

The relationship between the tRF-29 expression level in tissues and clinical parameters of AGC patients was calculated. Based on the tRF-29 level in tissue samples of AGC patients, we grouped the patients into low tRF-29 group (**−**Δ*C*q < −5.4242, *n* = 60) and high tRF-29 group (**−**Δ*C*q > −5.4242, *n* = 59). Our study revealed a significant correlation between tRF-29 levels and tumor differentiation and size (Table 1). The above results indicated that tRF-29 was related to the proliferative capacity of GC.

**Table 1:** The correlation between the amount of tRF-29-79MP9P9NH525 in tissues and clinicopathological features of advanced gastric cancer patients.

Characteristics	*n* (%)	High (%)	Low (%)	*P*-value
**All cases**	119	59	60	
**Gender**				>0.9999
Male	71 (59.66)	35 (59.32)	36 (60.00)	
Female	48 (40.34)	24 (40.68)	24 (40.00)	
**Age (y)**				0.1627
≥60	83 (69.75)	45 (76.27)	38 (63.33)	
<60	36 (30.25)	14 (23.73)	22 (36.67)	
**Differentiation**				0.0107
Well	9 (7.56)	5 (8.48)	4 (6.67)	
Moderate	68 (57.14)	41 (69.49)	27 (45.00)	
Poor	42 (35.30)	13 (22.03)	29 (48.33)	
**Lymphatic node metastasis**				>0.9999
N0	37 (31.09)	18 (30.51)	19 (31.67)	
N1 & N2 & N3	82 (68.91)	41 (69.49)	41 (68.33)	
**Invasion**				0.1563
T2	39 (32.77)	20 (33.90)	19 (31.67)	
T3	7 (5.88)	1 (1.69)	6 (10.00)	
T4	73 (61.35)	38 (64.41)	35 (58.33)	
**Tumor size (cm)**				0.0278
≥5	63 (52.94)	25 (42.37)	38 (63.33)	
<5	56 (47.06)	34 (57.63)	22 (36.67)	
**Distal metastasis**				0.3644
Yes	4 (3.36)	3 (5.08)	1 (1.67)	
No	115 (96.64)	56 (94.92)	59 (98.33)	
**TNM stage**				0.6373
IB	25 (21.00)	13 (22.03)	12 (20.00)	
IIA & IIB	24 (20.17)	10 (16.95)	14 (23.33)	
IIIA & IIIB & IIIC	66 (55.46)	33 (55.93)	33 (55.00)	
IV	4 (3.36)	3 (5.09)	1 (1.67)	
**Vessel invasion**				>0.9999
Yes	85 (71.43)	42 (71.19)	43 (71.67)	
No	34 (28.57)	17 (28.81)	17 (28.33)	
**Lymphatic invasion**				0.6883
Yes	84 (70.59)	43 (72.88)	41 (68.33)	
No	35 (29.41)	16 (27.12)	19 (31.67)	
**Nerve invasion**				0.0877
Yes	76 (63.87)	33 (55.93)	43 (71.67)	
No	43 (36.13)	26 (44.07)	17 (28.33)	
**Ki-67 (%)**				0.3689
>30	94 (78.99)	49 (83.05)	45 (75.00)	
≤30	25 (21.01)	10 (16.95)	15 (25.00)	

Furthermore, plasma tRF-29 also showed low level in the GC group (Fig. 2D), which was in accordance with its level in tissue samples. Next, we constructed the ROC curve based on tRF-29 level in pre-operative plasma and healthy controls. At the optimized cut-off value of -0.548, the AUC of plasma tRF-29 showed 0.746 (Fig. 2E), which was slightly lower than its value in tissues (Supplementary Fig. 2). However, when combined with traditional tumor markers, the diagnostic value of plasma tRF-29 was significantly improved. We found that the AUC was increased after the combination with CEA (AUC: 0.806, Fig. 2F), CA125 (AUC: 0.772, Fig. 2G), CA19-9 (AUC: 0.763, Fig. 2H), and AFP (AUC: 0.746, Fig. 2I). Meanwhile, the joint diagnostic value of five biomarkers was the best (AUC: 0.827, Fig. 2J).

To determine the prognostic significance of plasma tRF-29 in GC, we followed up the GC patients. However, eight patients were lost in follow-up investigation. The overall survival (OS) was calculated based on death as the clinical endpoint. The evaluation period for OS lasted approximately 27 months. Kaplan-Meier survival analysis indicated a potential correlation between low plasma tRF-29 levels and decreased OS rates (*P* = 0.0094, Supplementary Fig. 3A). Besides, using a multivariate Cox analysis, tRF-29 was validated as a powerful predictor in predicting an unfavorable outcome for patients with GC (Supplementary Fig. 3B). The results mentioned above revealed the diagnostic significance of tissue tRF-29 in GC and the potential of plasma tRF-29 in improving diagnostic efficiency in GC when combining with other tumor markers, as well as the prognostic values of plasma tRF-29 in GC.

### Effects of tRF-29-79MP9P9NH525 on cell viabilities in vitro

**Fig. 3:**
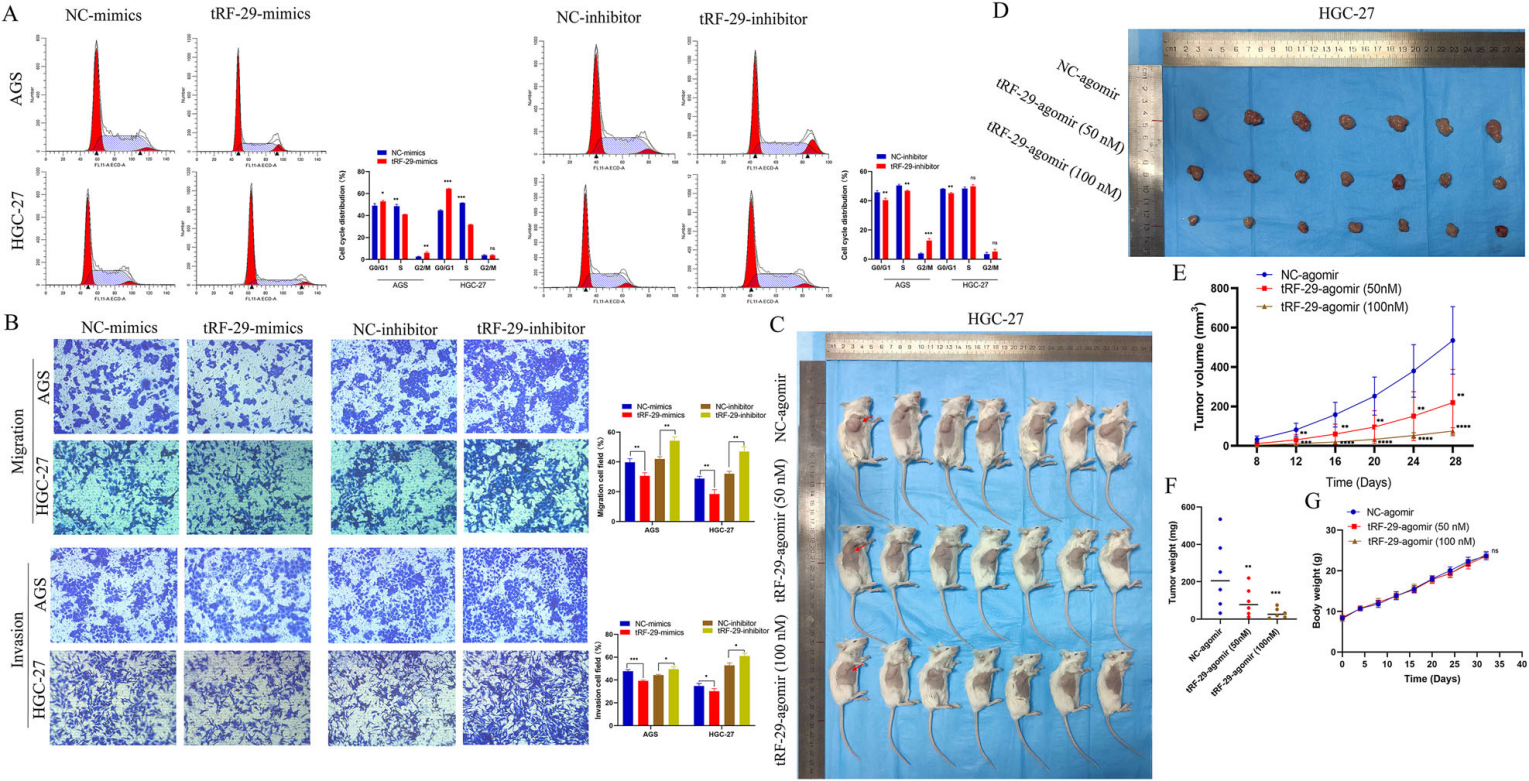
The effects of tRF-29-79MP9P9NH525 (tRF-29) on the growth and metastasis of gastric cancer (GC) in vitro and in vivo. **A** The effects of tRF-29 on cell cycle distribution of GC were determined by flow cytometry. *n* = 3. **B** tRF-29 demonstrated inhibitory effects on the migratory and invasive viability of AGS and HGC-27 cells. Conversely, the knockdown of tRF-29 resulted in the opposite effects. *n* = 3. **C** HGC-27 cells with different concentrations of tRF-29-agomir were hypodermically injected into the right axilla of mice. **D** Tumors obtained from mice after euthanized. **E** Tumor volume change curve. *n* = 7. **F** Tumor weight after being euthanized. *n* = 7. **G** The body weight of the mice. *n* = 7. Comparing with NC, *P < 0.05, **P < 0.01, ***P < 0.001, ****P < 0.01; ns no significance.

Based on the results of tRF-29 in tissue samples and plasma samples, and its relationship with clinical parameters, we wondered the biological roles of tRF-29 in GC. tRF-29 exhibited downregulation in gastric cancer AGS, HGC-27, and MKN-45 cells, relative to its expression in normal GES-1 cells, which pattern was consistent with its levels in tissue and plasma samples (Supplementary Fig. 4A). Since AGS (moderate differentiated) and HGC-27 (undifferentiated) are cells with different degrees of differentiation and represent different types of gastric cancer, they were used in the following studies. We succeed in upregulating or downregulating tRF-29 level in AGS and HGC-27 cells via tRF-29 mimics or tRF-29 inhibitor treatment, respectively. The efficiency of the regulated tRF-29 was verified by qRT-PCR (Supplementary Fig. 4B, C). The EdU and cell cloning assays revealed that the proliferation of AGS and HGC-27 cells was significantly restrained with tRF-29 overexpression, while decreasing tRF-29 promoted the proliferation (Supplementary Fig. 4D, E). Additionally, overexpression of tRF-29 resulted in more GC cells halting at G0/G1 phase comparing with NC group, while the GC cells with relatively low tRF-29 level reduced GC cells at G0/G1 phase (Fig. 3A). Concurrently, the impact of tRF-29 expression levels on cellular migration and invasion was evaluated utilizing a Transwell assay. The migratory and invasive potentials of the AGS and HGC-27 were diminished in the presence of the tRF-29 mimic, whereas they were enhanced upon treatment with the inhibitor (Fig. 3B).

We further explored the effects of tRF-29 on the growth of GC in vivo. HGC-27 cells were transfected with tRF-29 agomir at different concentrations (50 nM or 100 nM) or NC-agomir, and subcutaneously injected into the right axilla of immunocompromised mice (Fig. 3C). Tumor volume and weight measurements revealed a dose-dependent inhibition of GC proliferation by tRF-29 upregulation (Fig. 3D–F). The body weight of mice was not affected during tumor growth (Fig. 3G).

### tRF-29-79MP9P9NH525 regulates AKT/P27 pathway via targeting KIF14

**Fig. 4:**
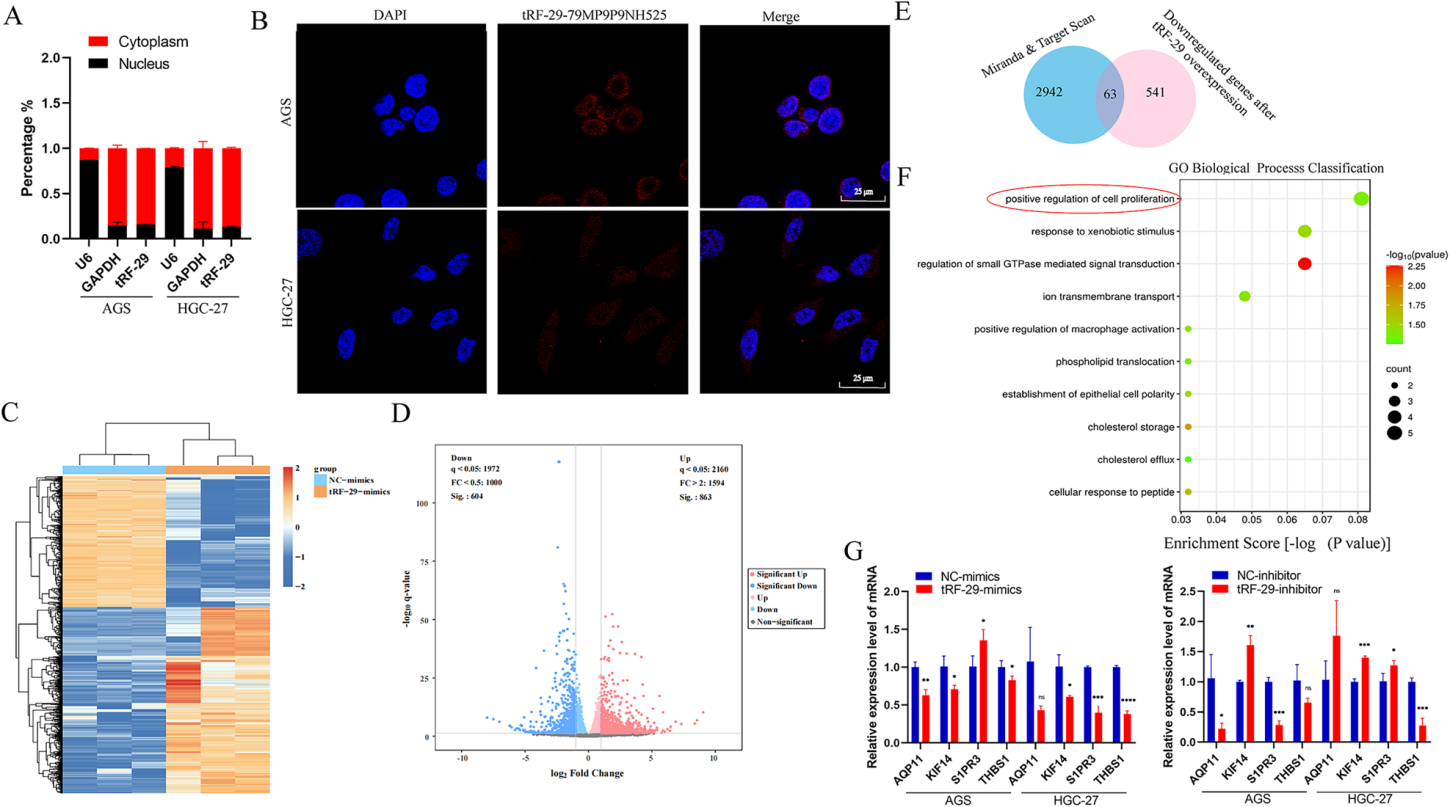
Exploring potential targeted genes of tRF-29-79MP9P9NH525 (tRF-29). **A**, **B** qRT-PCR results of nucleoplasmic separation (**A**) and FISH assay (**B**) identified the distribution of tRF-29 in gastric cancer cells. **C**, **D** The heatmap (**C**) and volcano plots (**D**) of the RNA sequence results in AGS cells treated by tRF-29 mimics. **E** The overlapped genes that were predicted by Miranda & TargetScan, and the downregualted genes in AGS cells treated by tRF-29 mimics. **F** The bubble diagram exhibited the biological process classification of tRF-29’s targeted genes. **G** The expression level of aquaporin 11 (AQP11), kinesin family member 14 (KIF14), sphingosine-1-phosphate receptor 3 (S1PR3), and thrombospondin 1 (THBS1) were detected in gastric cancer cells with tRF-29 upregulation or downregulation. *n* = 3; *P < 0.05, **P < 0.01, ***P < 0.001, ****P < 0.0001, ns no significance.

To elucidate the molecular mechanisms underlying tRF-29 regulating GC occurrence, the subcellular location of tRF-29 in GC cells was determined. Nucleoplasmic separation followed by qRT-PCR was performed. The results showed that the vast majority of tRF-29 were located in the cytoplasm of AGC and HGC-27 cells (Fig. 4A). The subcellular location of tRF-29 was further determined by FISH assay (Fig. 4B). The cytoplasmic location of tRF-29 led us to probe the potential roles of tRF-29 in a manner similar to those of microRNAs (miRNAs). As a result, RNA sequencing was then performed in GC cells with tRF-29 upregulation (SUB00040023, https://www.biosino.org/node/). It was found that 604 genes were significantly downregulated, and 863 genes were significantly upregulated after tRF-29 overexpression (Fig. 4C, D), respectively. To map the possible function of tRF-29 regulating downstream genes via targeting its 3′UTR, we performed Targetscan (http://www.targetscan.org/) and Miranda (http://www.microrna.org/microrna/home.do) prediction tools to predict the target genes of tRF-29. The intersection results of dry and wet experiments showed that a total of 63 genes that included complementary sequences with tRF-29 and were downregulated by tRF-29 overexpression were found (Fig. 4E). Subsequently, a Gene Ontology (GO) functional enrichment analysis was conducted on the identified genes. The results showed that aquaporin 11 (AQP11), kinesin family member 14 (KIF14), sphingosine-1-phosphate receptor 3 (S1PR3), and thrombospondin 1 (THBS1) were enrolled in the regulation of cell proliferation (Fig. 4F). Then, we performed qRT-PCR to detect their gene expression level in GC cells with tRF-29 alteration. Stimulatingly, KIF14 mRNA was found as the only gene that negatively correlated with tRF-29 levels change in GC cells (Fig. 4G).

**Fig. 5:**
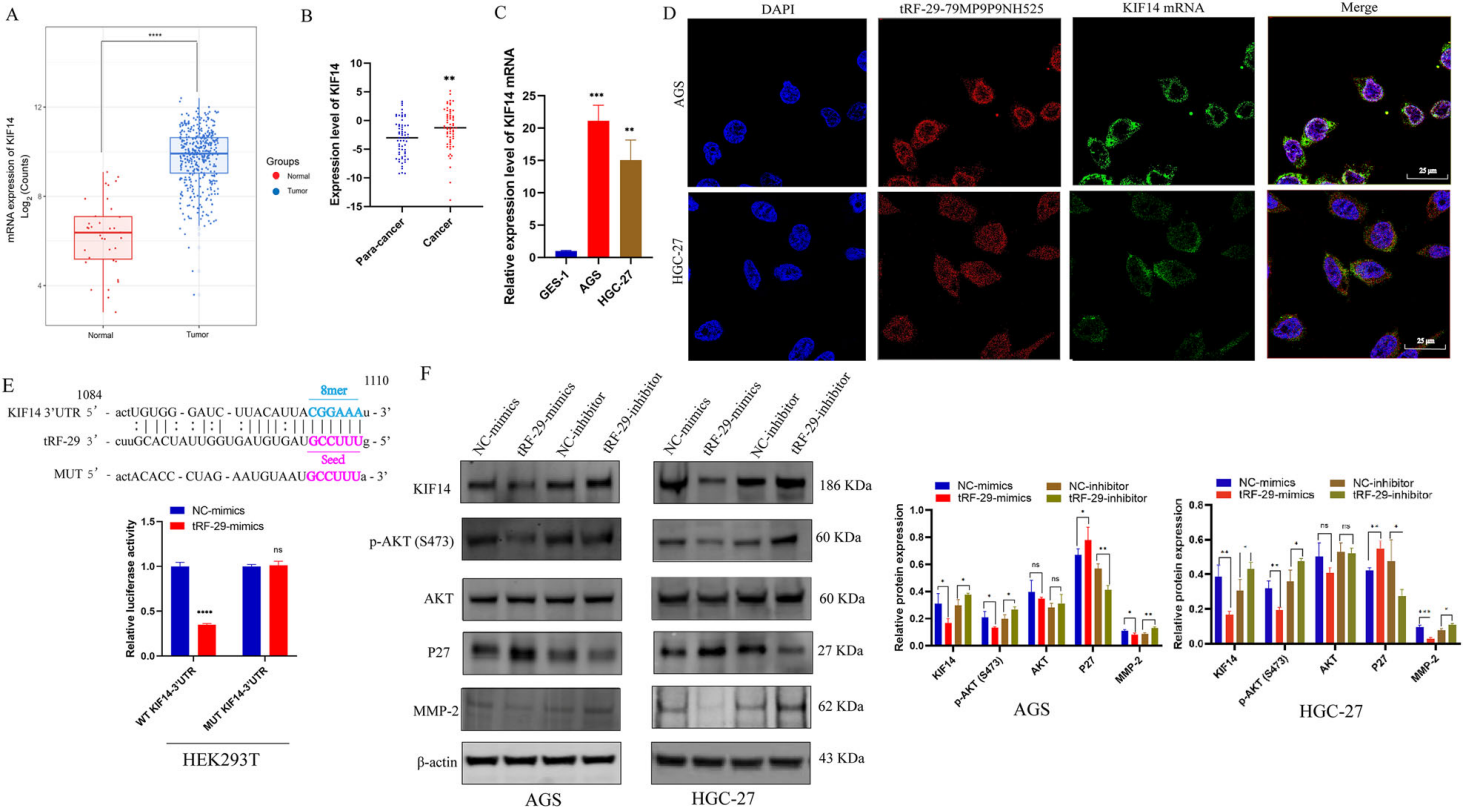
tRF-29-79MP9P9NH525 (tRF-29) regulates AKT pathway via silencing KIF14 expression. **A** The expression level of KIF14 in 413 cases of gastric cancer and 35 cases of healthy controls (Analyzed data from TCGA). **B** The expression level of KIF14 (−Δ*C*_q_) in paired advanced gastric cancer tissues and adjacent normal tissues was detected by qRT-PCR. *n* = 64. **C** KIF14 is upregulated in gastric cancer AGS and HGC-27 cells compared with GES-1 cells. *n* = 3. **D** The co-location of tRF-29 and KIF14 mRNA was detected by FISH. **E** The results of luciferase activity assay. *n* = 4. **F** tRF-29 overexpression reduced the protein level of KIF14, p-AKT(S473), and MMP-2, and increased the protein level of P27; while the protein level of AKT was not significantly changed. tRF-29 downregulation showed the reversed effects. *n* = 3. *P < 0.05, **P < 0.01, ***P < 0.001; ns no significance.

As a molecular motor involved in cytokinesis, KIF14 serves as oncogene in numerous malignancies including cholangiocarcinoma, prostate, cervical, and lung cancers [19–22]. We then accessed the data on KIF14 expression levels from The Cancer Genome Atlas (TCGA) database and observed a significantly high expression of KIF14 in GC group compared to that of healthy group (Fig. 5A). Further investigated the expression of KIF14 in 64 pairs of GC samples and their adjacent non-cancerous tissues (Fig. 5B), as well as its level in GES-1, AGS, and HGC-27 cells (Fig. 5C). The results showed that GC samples, GC cells exhibited higher level of KIF14, which imply the possible promoted effects of KIF14 in GC development.

To clarify the regulated mechanism of tRF-29 on KIF14 expression. FISH co-localization was performed. The results revealed that most tRF-29 and KIF14 mRNA were co-localized within the cytoplasm of GC cells (Fig. 5D). Then the possible binding sequences between tRF-29 and 3′UTR region of KIF14 mRNA was predicted (Fig. 5E). The above findings provide supports for the hypothesis that tRF-29 down-regulates KIF14 expression by targeting its 3′UTR. As a result, we first constructed wild type (WT) and mutational type (MUT) 3′UTR reporter plasmid of KIF14. Then HEK293T cells were co-treated with tRF-29 mimics or negative controls and reporter plasmid. The relative luciferase activity of the WT-KIF14-3′UTR was quenched by tRF-29 overexpression, while the mutated reporter plasmid disrupted the interaction of tRF-29 and 3′UTR of KIF14 (Fig. 5E). The results provided direct evidence for supporting our hypothesis.

**Fig. 6:**
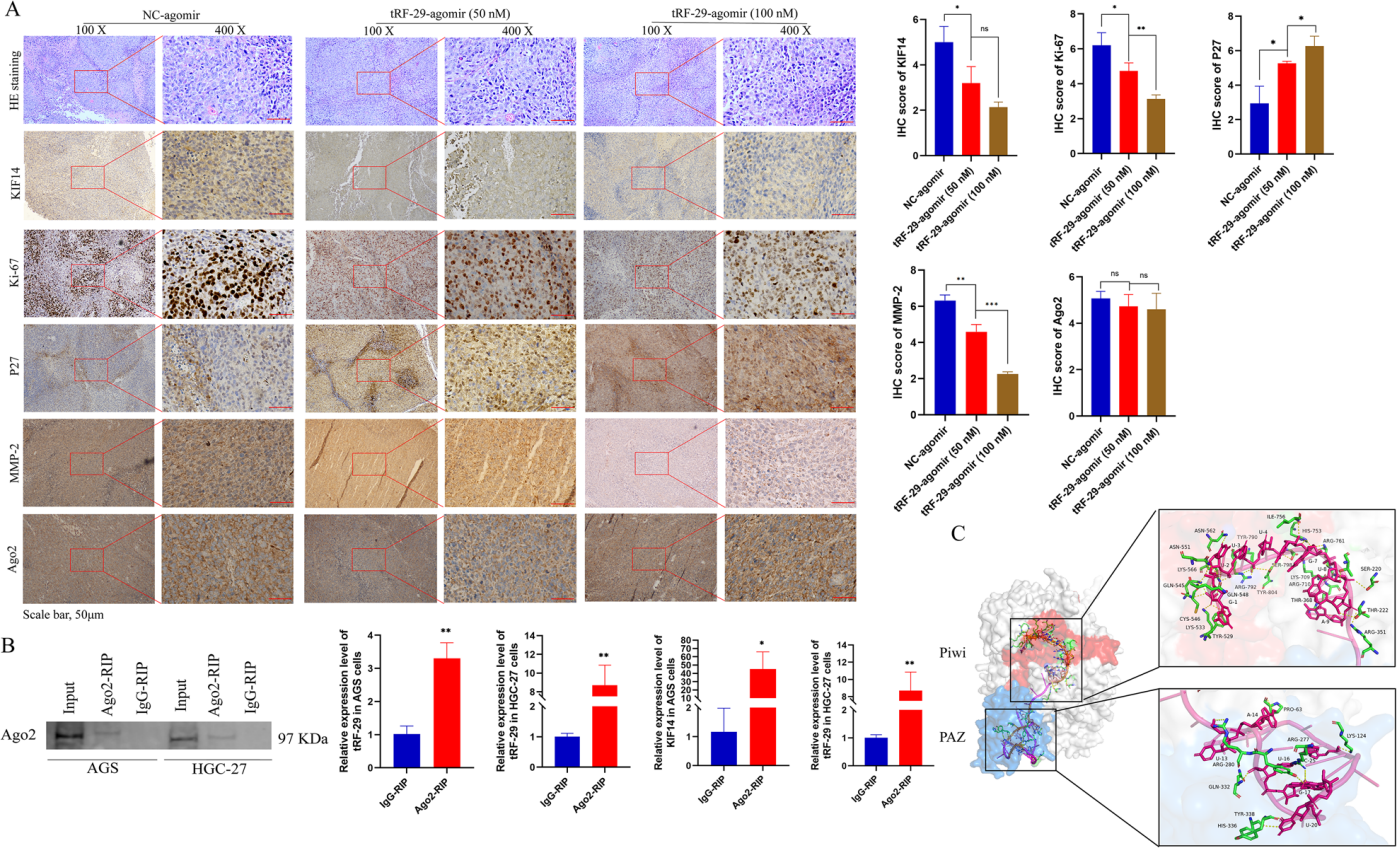
tRF-29-79MP9P9NH525 (tRF-29) silences KIF14 via incorporating with Ago2. **A** Immunohistochemistry (IHC) results of the protein levels of KIF14, Ki-67, P27, MMP-2, and Ago2 in tumor samples obtained from xenograft euthanized mice. *n* = 3. **B** tRF-29 and KIF14 mRNA were significantly enriched in Ago2-RIP group compared to IgG-RIP group. *n* = 3. **C** The binding between tRF-29 and the Ago2 protein. PIWI and Piwi-Argonaut-Zwille (PAZ) domains of Ago2 were two binding domains predicted by the online database HDOCK (http://hdock.phys.hust.edu.cn/). *P < 0.05, **P < 0.01, ***P < 0.001; ns no significance.

Previous studies reported that KIF14 increased the activity of AKT (also named as protein kinase B, PKB) by phosphorylation of AKT [21, 23]. Here we wondered whether tRF-29-mediated attenuation of KIF14 also regulated the activity of AKT, as well as its downstream effector molecule P27 and MMP-2 [24, 25]. The results revealed that tRF-29 overexpression damped KIF14 expression, and subsequently reduced levels of AKT phosphorylation and MMP-2, while P27 was found to be upregulated as the result of p-AKT downregulation (Fig. 5F). Contrarily, tRF-29 knockdown resulted in upregulation of KIF14, and improved levels of AKT phosphorylation and upregulated the expression of MMP-2, while the levels of P27 were reduced (Fig. 5F). Furthermore, IHC staining in HGC-27 xenograft tumors was conducted to determine tRF-29’s effects on KIF14. The findings indicated that upregulation of tRF-29 in tumors was associated with downregulation of KIF14 expression. Simultaneously, the levels of P27 were upregulated while the levels of MMP-2 were reduced. The modulated effects of tRF-29 on KIF14, P27, and MMP-2 showed dose-dependent manner (Fig. 6A). We also wondered whether there was an effect of tRF-29 on KIF14/AKT pathway in human tissue samples. We detected the MMP-2, p-AKT(S473), and P27 expression levels in advanced gastric cancer tissues and adjacent normal tissues with the IHC method. The results showed that para-carcinoma tissues with tRF-29 upregulation exhibited low expression of KIF14, MMP-2, and p-AKT(S473), and high expression of P27; while in cancer tissues with tRF-29 downregulation showed significantly high expression of KIF14, MMP-2, and p-AKT(S473), and low level of P27 (Supplementary Fig. 5). The results indicated that KIF14 regulated GC cell growth and migration via regulating AKT signaling pathway.

Several studies reported that tRFs confer gene silencing by binding with Ago, which is a critical component of RNA-induced silencing complex (RISC) [26, 27]. To map the post-transcriptional silencing of tRF-29 via forming RISC, we performed Ago2-RIP assay in AGS and HGC-27 cells. RNA precipitated by anti-Ago2 and anti-IgG was purified and subjected to qRT-PCR. The outcomes indicated that Ago2 exhibited significantly higher enrichment of tRF-29 compared to IgG (Fig. 6B), which indicates the interaction between tRF-29 and Ago2 protein. The online database HDOCK (http://hdock.phys.hust.edu.cn/) provided insight into the binding pattern between tRF-29 and Ago2. We found that PIWI and Piwi-Argonaut-Zwille (PAZ) domains of Ago2 were pivotal in binding to tRF-29 (Fig. 6C). The above-mentioned results indicated that tRF-29 interacted with Ago2 to assemble silencing complexes. Moreover, qRT-PCR of KIF14 followed by RIP demonstrated that Ago2 could substantially enrich KIF14 mRNA (Fig. 6B). We also wondered whether there was any effect on the Ago2 expression after inhibition or enhancement of tRF-29. WB and qRT-PCR were performed in AGS and HGC-27 cells with tRF-29 overexpression or downregulation. The results showed no obvious effects of tRF-29 on Ago2 mRNA expression or protein level (Supplementary Fig. 6A, B). tRF-29 treated HGC-27 xenograft tumors also showed no changes of Ago2 level (Fig. 6A).

### KIF14 attenuation reverses tRF-29-79MP9P9NH525 inhibition mediated effects on GC cells

**Fig. 7:**
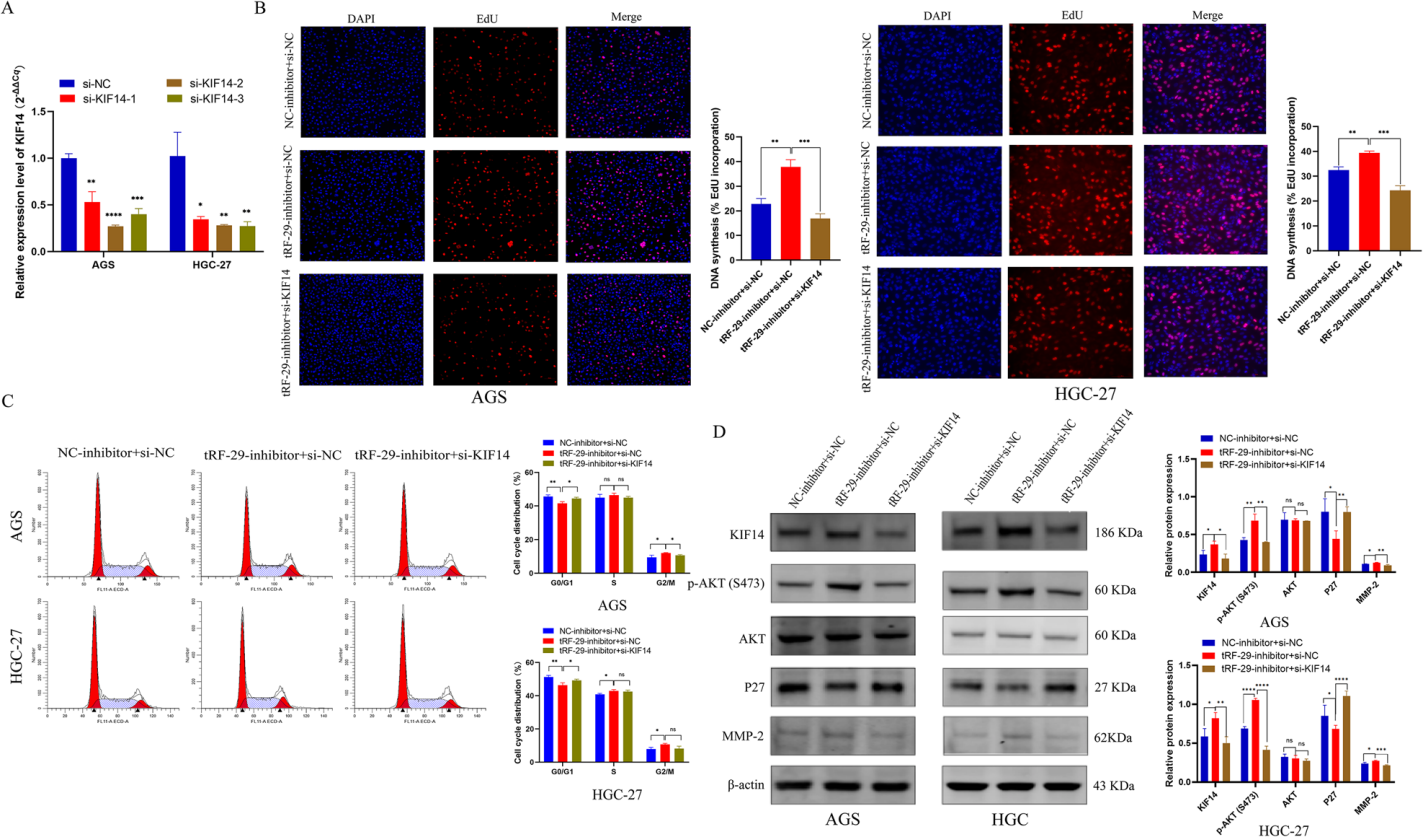
Reduced KIF14 expression reversed the effects of tRF-29-79MP9P9NH525 (tRF-29) on the proliferation of gastric cancer. **A** The downregulated effect of si-KIF14 was determined by qRT-PCR. *n* = 3. **B** EdU assay revealed the rescue effects of KIF14 knockdown in cells treated by tRF-29 inhibitor. *n* = 3. **C** Reduced KIF14 expression trapped more cells in G0/G1, which was reduced by tRF-29 inhibitor. *n* = 3. **D** The activated p-AKT(S473) pathway by tRF-29 inhibitor was restrained by KIF14 downregulation. *n* = 3. *P < 0.05, **P < 0.01, ***P < 0.001, ****P < 0.0001; ns no significance.

To verify the effects of tRF-29 on GC were regulated by KIF14 level, rescue experiments were performed. First, three different siRNAs targeted KIF14 mRNA on were designed, then the knockdown efficiency was assessed using qRT-PCR (Fig. 7A). We reduced KIF14 mRNA levels in GC cells with tRF-29 downregulation. Our study revealed that the inhibition of KIF14 resulted in the suppression of the stimulatory effects of tRF-29 on the proliferation and cell cycle progression of GC cells (Fig. 7B, C). In the migration and invasive experiments, we also found that the inhibition of KIF14 led to a reduction in the promoted effects of tRF-29 on cellular metastasis (Supplementary Fig. 7). WB results further showed the increased level of AKT phosphorylation and MMP-2, while P27 was downregulated in GC cells with tRF-29 overexpression and KIF14 downregulation (Fig. 7D). The results demonstrated that tRF-29 regulates GC proliferation and metastatic viability through modulating KIF14 expression.

## Discussion

In China, GC ranks third in both incidence and mortality, with over 4.5 million newly diagnosed cases and approximately over 3 million cancer-related death in 2020 [28]. The widespread adoption of gastroscopy has led to a modest increase in the 5-year survival rate of gastric cancer to approximately 36% [28]. The poor prognosis of GC is partially related to diagnosis at advanced stage, which is partially related to the low efficiency of early screening and the lack of sensitive biomarkers. It is urgently needed to develop novel biomarkers to improve GC screening efficiency.

As the progression of gastric cancer involves a sequential development from BL to PL, followed by EGC, and eventual AGC. Are molecular biomarkers different in various stages of gastric mucosa pathology? Presently, we endeavored to detect molecular biomarkers that are sensitive to different stages of stomach lining. Based on high-throughput sequencing of tRNA-derived small RNA (tsRNA) in plasma from healthy donors and GC patients [18], we identified tRF-29 as GC-related tsRNA and constructed a specific qRT-PCR detection method for tRF-29 quantification in tissue and plasma samples. Thanks to the design of stem-loop RT primer, which was effective in extending the cDNA template (Supplementary Fig. 1B), tRF-29 that was only 29 nt was successfully detected. Through training and validation sets, tRF-29 was found to be downregulated in tissue samples from GC patients (Fig. 2A, B). To figure out whether the tRF-29 level in different gastric mucosal stages was changed, we collected various gastric mucosal tissue samples. To our surprise, tissue tRF-29 level showed a gradient change among HCs, BL, PL, EGC, and AGC groups (Fig. 2C). By ROC construction between different cohorts, we found that tRF-29 showed a higher value in discriminating AGC and EGC from HCs, BL, and PL (Supplementary Fig. 2). In addition, tRF-29 also showed an excellent value in distinguishing PL from HCs (Supplementary Fig. 2), which suggests the potential significance of tRF-29 as a marker for monitoring various types of gastric mucosal conditions.

The diagnostic value of tissue tRF-29 in GC encouraged us to evaluate its value in plasma samples of GC patients. As expected, tRF-29 also showed a lower level in pre-operative plasma than that of post-operative plasma of GC patients and healthy controls (Fig. 2D). Plasma tRF-29 showed a slightly low value in distinguishing GC from HCs. However, when combined with CEA, CA125, CA19-9, and AFP, its diagnostic value was significantly improved (Fig. 2E–J). The results imply that novel molecular markers may improve the diagnostic value of common tumor markers for GC. Furthermore, we followed up patients with GC who were subject to plasma tRF-29 detection and took about 27 months for OS to be evaluated. Strikingly, tRF-29 emerged as a powerful and independent prognostic indicator of poor OS of GC patients (Supplementary Fig. 3).

To study the tRF-29′s biological roles in GC development, we evaluated the association of tissue tRF-29 and corresponding AGC patients′ clinical parameters. It was found that reduced tissue tRF-29 was correlated with bigger tumor size of AGC (Table 1). The results presented above signify that tRF-29 may related to GC progression. In the cellular function experiments, we found that tRF-29 overexpression resulted in reduced growth, subdued migration and invasion ability of AGS and HGC-27 cells (Fig. 3B; Supplementary Fig. 4D, E); Flow cytometry showed that upregulation of tRF-29 arrested more cells in G0/G1 stage (Fig. 3A); while tRF-29 downregulation promoted the proliferation, migratory and invasive capacity of GC cells, and reduced the cells in G0/G1 stage. Moreover, the inhibitory effects of tRF-29 on the proliferation of GC cells were also validated in the xenograft tumor formation assay (Fig. 3C–F). The experiments conducted in cellular and mice models promoted further investigation into the molecular mechanism by which tRF-29 regulates the progression of GC. Several studies reported that tsRNA exerted their regulation roles by binding with RNA binding protein (RBP) or by targeting downstream mRNA [11, 29]. It is widely accepted that most tsRNAs function in a way similar to miRNA [30, 31]. In the aforementioned assays, we found that tRF-29 levels′ change caused changes in KIF14 levels, there’s necessity in disclosing the underlying mechanism. Intriguingly, RNA-FISH found that tRF-29 and KIF14 mRNA were co-located in AGS and HGC-27 cells (Fig. 4A, B), we assumed that tRF-29 functions via silencing some oncogenes. With the combined application of transcriptome sequencing in AGS cells with tRF-29 overexpression (Fig. 4C, D), we found that 63 predicted genes contain complementary sequences with tRF-29 (Fig. 4E). GO enrichment analysis revealed that the most target genes of tRF-29 were enrolled in the regulation of cell proliferation (Fig. 4F). We first selected genes that were enrolled in the regulation of cell proliferation and further performed qRT-PCR to detect their expression change in GC cells with tRF-29 alteration. Among them, the level of KIF14 mRNA was the most correlated with tRF-29 level changes (Fig. 4G).

KIF14, as a member of the kinesin family, plays a role in the intracellular transportation of cargo vesicles, the formation of mitotic spindles, the separation of chromosomes, intermediate formation, and completion of cytokinesis [32]. Kinesin family proteins are broadly regarded as potential targets for cancer therapies due to their involvement in tumorigenesis and cancer progression [19, 22]. Studies reported the carcinogenic effects of KIF14 in several cancers. For example, KIF14 was found to promote the proliferation and lymphatic metastasis of cholangiocarcinoma [19]. In GC, elevated level of KIF14 was reported to facilitate tumor growth and correlated with advanced tumor stage, tumor-node-metastasis (TNM) classification, and the presence of metastasis [23]. In our study, we excavated data of KIF14 in TCGA, KIF14 exhibited higher level in GC tissues than normal controls (Fig. 5A). In 64 pairs of AGC tissue samples and paired normal tissues obtained by our group, as well as GC cell lines, we also observed a significant upregulation of KIF14 in gastric cancer tissues and cell lines (Fig. 5B, C), which imply that KIF14 may serve as oncogene in GC development. In several studies, the potential mechanisms of KIF14 in malignant viability were reported. For example, KIF14 was found to contribute to chemoresistance in advanced prostate cancer through the activation of AKT. Zhang et al. found that KIF14 inhibited cervical cancer cell cycle progression by influencing the level of cyclin-dependent kinase inhibitor (CDKI) P27 [22]. The dual luciferase reporter assay determined that tRF-29 inhibited wild-type KIF14 mRNA luciferase activity via targeting its 3′-UTR (Fig. 5D, E), which implied the possible mechanism of tRF-29 in suppressing GC progression via regulating KIF14.

Taking into account KIF14’s role in activating AKT pathway, we hypothesized that tRF-29 regulated cell proliferation and migration of GC by modulating AKT pathway. In the current study, we found that increased tRF-29 resulted in reduced level of KIF14, phosphorylation of AKT, and MMP-2, and upregulation of P27; while reduced tRF-29 showed the reversed effects (Fig. 5F). Moreover, the regulated effects of tRF-29 on KIF14/AKT axis were also determined in the xenograft tumor assay. The IHC results showed that tRF-29 upregulation resulted in attenuated activity of AKT, reduced KIF14 and MMP-2 expression, and upregulated P27 expression (Fig. 6A). Ki-67, a famous proliferation marker, further confirmed the tRF-29’s suppressor role on GC (Fig. 6A). To verify tRF-29’s influence on KIF14/AKT pathway, we detected protein levels of KIF14, MMP-2, p-AKT(Ser473), and P27 in human tissue samples via IHC. The results exhibited that KIF14, p-AKT(Ser473), and MMP-2 showed high level, while P27 showed low level in GC tissues with tRF-29 downregulation (Supplementary Fig. 5B). Conversely, in normal tissues with tRF-29 upregulation, we found the low expression of KIF14, p-AKT(Ser473), and MMP-2, and high expression of P27 (Supplementary Fig. 5B).

For further determination of tRF-29’s effects on the proliferation and metastasis of GC via regulating KIF14, we applied cells’ viability rescue experiments. KIF14 downregulation reversed the effect of tRF-29’s effects on cells proliferation, migration, and invasion (Fig. 7B, C, Supplementary Fig. 7). The protein levels of p-AKT, MMP-2, and P27 provided additional confirmation that the downregulation of KIF14 reversed the stimulatory effects of the tRF-29 inhibitor on GC cells (Fig. 7D).

As a kind of small RNAs, tRFs were found to exert gene destabilization by binding with Ago to form RNA silencing complexes [33]. Moreover, distinct Ago subtypes show distinct affinities to different tRFs [34]. Ago2 was reported as the preferred Ago guiding tRFs to target genes [27, 35]. Green et al. proposed that IL-1β-induced tRF-3003a targets Janus Kinase (JAK) mRNA by binding to Ago2 in osteochondrocytes [27]. For confirmation of the interaction between tRF-29 and Ago2 in the present study, RIP assay found that Ago2 bound to tRF-29 and KIF14 mRNA (Fig. 6B). HDOCK was further used to predict the critical domains for interaction. Intriguingly, PIWI and PAZ domains were predicted as pivotal domains in interacting with tRF-29 (Fig. 6C). The PIWI domain is a conserved domain present in Piwi proteins and numerous other nucleic acid-binding proteins, particularly those involved in RNA binding and cleavage [36]. The PAZ domain is present in the Piwi and Dicer protein families, which are crucial components of RNA-mediated gene-silencing pathways [37]. We examined whether altering tRF-29 levels affects Ago2 expression in GC cells and xenograft tumors. The results show no significant differentiated expression of Ago2 in xenografts or GC cells with the difference in tRF-29 level (Fig. 6A, Supplementary Fig. 6A, B). The aforementioned results signified that tRF-29 binds with Ago2 without influencing Ago2 stability and inhibited KIF14 expression.

## Conclusion

Our findings implicate that tRF-29-79MP9P9NH525 (tRF-29) might be promising biomarker for GC diagnosis and prognosis evaluation, and function as a potential monitor in tracing pathological status of gastric mucosa. In terms of mechanism, tRF-29 played the tumor suppressor role in GC by silencing KIF14 expression and then modulated the AKT pathway.

## Patients and methods

### Tissue and plasma sample collection

Plasma and tissue sample collection and usage were performed according to the relevant stipulations of Human Research Ethics Committee of Ningbo University (No. 2019022501). Prior to the commencement of the research study, all participants provided written informed consent. The inclusion criteria required that patients were between the ages of 18 and 85, and that the diagnosis of gastric mucosal lesions was based on pathological examination. However, patients diagnosed with severe cardiovascular, cerebrovascular, and pulmonary diseases, or have previously undergone treatments including chemotherapy, radiotherapy, targeted therapy, immuno-therapy before surgical excision were excluded. The sample size was selected based on related literature regarding the impact of specific genes on disease diagnosis and prognosis [38]. A total of 532 tissue samples and 256 plasma samples were included. Tissue samples includes 172 pairs of advanced gastric cancer (AGC) and adjacent normal tissues (53 pairs tissues of AGC and adjacent normal tissues for training, 119 pairs tissues of AGC and adjacent normal tissues for validation), 47 tissues of healthy controls (HCs), 47 tissues of benign lesion (BL), 48 tissues of precancerous lesion (PL), and 46 tissues of early gastric cancer (EGC). The plasma samples included in this study comprised 88 samples from healthy controls, and 84 pairs of pre- and post-operative plasma samples from patients with GC. A quantitative real-time reverse transcription-polymerase chain reaction (qRT-PCR) system was constructed to detect the abundance of tRF-29. All patients were allocated into different groups based on histopathological examination. Following excision from the patient, tissue specimens were promptly preserved in RNAfixer Reagent (Cwbio, Jiangsu, China) and subsequently stored at –80 °C.

### Total RNA extraction, reverse transcription and PCR detection

RNA was isolated from plasma and tissues/cells were isolated via using TRIzol LS and TRIzol (Invitrogen, Carlsbad, CA, USA), respectively. For the detection of tRF-29, Polestar 1st cDNA Synthesis Kit (Tiosbio, Beijing, China) was used for reverse transcription, while KOD SYBR qPCR Mix Kit (Toyobo, Tokyo, Japan) was used for qRT-PCR. The specific hairpin reverse transcription (RT) primer, upstream and downstream primers for the detection of tRF-29 were designed and listed in Supplementary Table 1. Plasma and tissue samples were assessed using −Δ*C*_q_ method, and the relative expression in cells was assessed using 2^−ΔΔ*C*q^ method with reference to small nuclear RNA U6 [16]. GoScript reverse transcription system and GoTaq qPCR Master Mix (Promega, Madison, WI, USA) were employed for mRNA quantification, which were normalized by glyceraldehyde-3-phosphate dehydrogenase (GAPDH) mRNA.

### Cell culture and foreign genetic materials transfection

The study includes the normal human gastric mucosal epithelium cell line GES-1 and human GC cell lines, AGS, HGC-27, and MKN-45. All cells were obtained from Cell Resource Center of Shanghai Institutes for Biological Sciences, Chinese Academy of Sciences, China. Roswell Park Memorial Institute (RPMI) 1640 (HyClone, Los Angeles, CA, USA) supplemented with 10% fetal bovine serum (FBS) (PAN-Biotech, Aidenbach, Germany) was used to cultivate all cells in an incubator at 37 °C and 5% CO_2_. Exogenous tRF-29 mimic/inhibitor, and corresponding negative control (NC) (Supplementary Table 1) were constructed (GenePharma, Shanghai, China) and transferred at the optimal concentration. Three distinct small interfering RNAs (siRNAs), namely si-KIF14-1, si-KIF14-2, and si-KIF14-3 were applied for knocking down KIF14 expression (Supplementary Table 1). Cells were subjected to experiments 48 h post-transfection.

### Cell proliferation assays

In the colony formation, treated cells were seeded (at a density of 500 cells/mL) in per well of six-well plates. After culturing for about 10 days, visible cloning spots appeared. The stained cloning spots were pictured and counted in Photoshop software (Adobe, San Jose, CA, USA) after fixing and staining. For the ethynyl-2′-deoxyuridine (EdU) experiment, cells were first cultivated with EdU solution (20 μM) for 2 h or more, then cells were fixed and undergo permeabilization with permeable solution (P0097, Beyotime, Shanghai, China) for 15 min, respectively. Later, the cells were subjected for staining reaction. Finally, the cell nucleus was stained with 1 × Hoechst33342 (Beyotime). The fluorescent microscope (Nikon Ds-Ri2, Tokyo, Japan) was used for imaging.

### Cell migration and invasion assays

For the migration assay, the 12-well plate was added with 500 μL of complete culture medium. During the invasion assay, the Transwell chambers were coated with Matrigel (Costar, Corning, NY, USA) before seeding cells. The 200 μL of cell medium without FBS mixing with 3 × 10^4^ cells were loaded into the upper chamber. Following 48-hour incubation, the traversed cells were stained and imaged.

### Flow cytometry analysis

Cells were synchronized by using serum-free medium before transfection. Then the transfected cells were fixed with 75% ethanol solution at −20 °C for at least 24 h. Before DNA staining, cells were hydrated with distilled water. After co-incubation DNA staining for 30 min with light avoidance, cells transferred to flow tubes for detection with flow cytometry (Epics Altra II; Beckman Coulter, Fullerton, CA, USA).

### Fluorescence in situ hybridization (FISH)

Cells were seeded in culture dishes with glass bottoms. After attachment to the glass sheets, cells were fixed and permeabilized. For the subcellular location of tRF-29, cells were hatched with Cy3 labeled tRF-29 probe, or co-incubated with tRF-29 probe and FAM-labeled KIF14 mRNA probe. Then 4’,6-diamidino-2-phenylindole (DAPI) (GenePharma) solution was used for cellular nuclei staining. Finally, the probe signals were captured by confocal microscopy (Leica, Heidelberg, Germany).

### Dual luciferase reporter assay

Bioinformatics predicted the KIF14 mRNAʹs complementary sequence with tRF-29. Then luciferase reporter plasmid was created with wild KIF14-3’ untranslational region (UTR) sequence (5ʹ-AAGTAATAAAGATAATATTCTACTTGTGGGATCTTACATTACGGAAATAGTTTGACGTTTTTGACCTCAA-3ʹ) or mutational KIF14-3’UTR sequence (5ʹ-AAGTAATAAAGATAATATTCTACTACACCCTAGAATGTAATGCCTTTAAGTTTGACGTTTTTGACCTCAA-3ʹ) was inserted. Human embryonic kidney HEK293T cells were co-treated with tRF-29 mimics or mimics control, and pmirGLO-KIF14-3’UTR-WT or pmirGLO-KIF14-3’UTR-MUT. After 48 h, detection of luciferase signals was conducted using the Dual Luciferase Assay Kit (Promega).

### Western blot (WB)

The indicated cells were collected by scraping, from which proteins were extracted by radioimmunoprecipitation assay lysis buffer (RIPA) lysate (Solarbio, Beijing, China). The protein concentration was detected by Bicinchoninic Acid (BCA) kit (Beyotime). The extracted proteins were separated in the gels soaking in running buffer by electrophoresis, and then transferred to polyvinylidene fluoride (PVDF) membrane (Millipore, Billerica, MA, USA). Subsequently, the membranes were obstructed using a blocking buffer (Jumpingfrog, Shanghai, China) for primary antibody (anti-KIF14, A10275, ABclonal, Wuhan, China; Phospho-Akt (Ser473), 4060, CST, MA, USA; anti-P27, 25614-1-AP, Proteintech, Wuhan, China; anti-AKT, A11016, ABclonal; anti-β-actin, AC028, ABclonal; anti-MMP-2, 10373-2-AP, Proteintech; anti-Ago2, ab186733, Abcam, Cambridge, UK) incubation. The secondary antibody incubation remained for 1–2 h at ambient temperature. The membranes were finally scanned and target proteins were visualized using the Odyssey laser imaging system (LI-COR Biosciences, Cambridge, UK).

### RNA-binding protein immunoprecipitation (RIP)

RNA Immunoprecipitation Kit (Millipore, Bedford, MA, USA) was used for RNA-binding protein immunoprecipitation. In short, RNA lysate of HGC-27 or AGS cells was lysed with RIP lysis buffer; the RNA supernate was collected and stored in −80 °C until the next procedure. The anti-IgG (Millipore) or anti-Ago2 (ab186733, rabbit monoclonal antibody; Abcam) was integrated with magnetic beads at room temperature; then antibody bound beads were incubated with RNA lysate at 4 °C overnight. The protein bound with beads was eluted for WB. Following extraction and purification, RNA was detected using qRT-PCR.

### Tumorigenicity test in vivo

For the sample size of nude mice, referring to a similar study on tumorigenicity in nude mice [14], 21 NOD-SCID male mice of 3-week old was used. The mice were kept in the Laboratory Animal Center of Ningbo University and cultured under the same feeding conditions. The mice were randomly grouped into a negative control group and tRF-29 overexpressing groups with 50 nM and 100 nM tRF-29-agomir, respectively. HGC-27 cells were first treated with NC-agomir or tRF-29-agomir (GenePharma), and subcutaneously injected into the right underarm of immunodeficient mice. The mice were sacrificed four weeks later for tumor resection and measurement.

### Immunohistochemistry (IHC) and H & E staining

Tumors resected from mice were fixed, embedded, and then were cut into 3 μm thickness slices. After deparaffinization in xylene, slices were rehydrated with a descending concentration of ethanol. After which, the slices were stained with hematoxylin and eosin (H & E) dye for structural identification of tumors. For the levels of KIF14, Ki-67, and P27 in xenograft tumors. Slices were subjected for antigen retrieval, and incubated with Anti-Ki-67 (27309-1-AP, Proteintech), Anti-KIF14 (26000-1-AP, Proteintech), Anti-P27 (25614-1-AP, Proteintech), Anti-p-AKT(Ser473) (4060, CST, MA, USA), Anti-MMP-2 (10373-2-AP, Proteintech) or anti-Ago2 (ab186733, Abcam) overnight at 4 °C. The following day, the slices were incubated with second antibody (SA00004-11, Proteintech) for 1 h at ambient temperature. Then, diaminobenzidine tetramine hydrochloride (DAB) reagent (Servicebio, Guangzhou, China) and hematoxylin staining (Solarbio, Beijing, China) were used to stain the slices. After sealing with neutral balsam (Solarbio), images were captured with microscopic inspection (Olympus, Tokyo, Japan).

### Statistical analysis

The analyses were conducted using GraphPad Prism v8.0 (San Diego, CA, USA) and SPSS Statistics Version 18.0 (Chicago, IL, USA). Data were presented as mean ± SD and analyzed using two-sided Studentʹs *t* test to compare groups. For small sample sizes (*n* < 5), plot individual points were described for data presentation. The receiver operator characteristic (ROC) curve was constructed to determine the diagnostic value. A Kaplan–Meier curve was used to assess the association between tRF-29 levels and GC patients′ survival, while multi-factor Cox regression was used to evaluate tRF-29’s prognostic value. A *P* value less than 0.05 was deemed to be statistically significant.

## Supplementary information

Supplementary data

Raw data of WB

## Data Availability

The datasets utilized and/or examined in the present investigation can be acquired from the author (lhlzhangshuangshuang@nbu.edu.cn) who provided them upon an acceptable request.
