# Correction to: Identification of tRF-29-79MP9P9NH525 as a biomarker and tumor suppressor of gastric cancer via regulating KIF14/AKT pathway

**DOI:** 10.1038/s41420-025-02614-6

**Published:** 2025-07-30

**Authors:** Jiaxin Ge, Ji Dai, Haoqiang Ji, Jie Guo, Xiaoban Shen, Desen Sun, Qiang Chen, Pan Chen, Guoliang Ye, Junming Guo, Shuangshuang Zhang

**Affiliations:** 1 Department of Gastroenterology, The First Affiliated Hospital of Ningbo University, 315020 Ningbo, China; 2 Department of Biochemistry and Molecular Biology, School of Basic Medical Sciences, Health Science Center, Ningbo University, 315211 Ningbo, China; 3 The First Department of General Surgery, Ningbo Zhenhai People’s Hospital, 315202 Ningbo, China; 4 School of Materials Science & Chemical Engineering, Ningbo University, 315211 Ningbo, China; 5 Ningbo Institute of Innovation for Combined Medicine and Engineering, The Affiliated Lihuili Hospital of Ningbo University, 315100 Ningbo, China

**Keywords:** Cell biology, Cancer

Correction to: *Cell Death Discovery* 10.1038/s41420-025-02514-9, published online 15 May 2025

In this article on page 4, lines 17 and 18 of Table 1: The correct number of “NO” situation in the high group is “18”; the correct number of “N1 &N2&N3” situation in the high group is “41”; the correct number of “NO” situation in the low group is “19”; the correct number of “N1&N2&N3” situation in the low group is “41”. The percentage and the total number (n) in the column 2 have been corrected accordingly.

**Table 1:** The correlation between the amount of tRF-29-79MP9P9NH525 in tissues and clinicopathological features of advanced gastric cancer patients.

Characteristics	*n* (%)	High (%)	Low (%)	*P*-value
**All cases**	119	59	60	
**Gender**				>0.9999
Male	71 (59.66)	35 (59.32)	36 (60.00)	
Female	48 (40.34)	24 (40.68)	24 (40.00)	
**Age (y)**				0.1627
≥60	83 (69.75)	45 (76.27)	38 (63.33)	
<60	36 (30.25)	14 (23.73)	22 (36.67)	
**Differentiation**				0.0107
Well	9 (7.56)	5 (8.48)	4 (6.67)	
Moderate	68 (57.14)	41 (69.49)	27 (45.00)	
Poor	42 (35.30)	13 (22.03)	29 (48.33)	
**Lymphatic node metastasis**				>0.9999
N0	37 (31.09)	18 (30.51)	19 (31.67)	
N1 & N2 & N3	82 (68.91)	41 (69.49)	41 (68.33)	
**Invasion**				0.1563
T2	39 (32.77)	20 (33.90)	19 (31.67)	
T3	7 (5.88)	1 (1.69)	6 (10.00)	
T4	73 (61.35)	38 (64.41)	35 (58.33)	
**Tumor size (cm)**				0.0278
≥5	63 (52.94)	25 (42.37)	38 (63.33)	
<5	56 (47.06)	34 (57.63)	22 (36.67)	
**Distal metastasis**				0.3644
Yes	4 (3.36)	3 (5.08)	1 (1.67)	
No	115 (96.64)	56 (94.92)	59 (98.33)	
**TNM stage**				0.6373
IB	25 (21.00)	13 (22.03)	12 (20.00)	
IIA & IIB	24 (20.17)	10 (16.95)	14 (23.33)	
IIIA & IIIB & IIIC	66 (55.46)	33 (55.93)	33 (55.00)	
IV	4 (3.36)	3 (5.09)	1 (1.67)	
**Vessel invasion**				>0.9999
Yes	85 (71.43)	42 (71.19)	43 (71.67)	
No	34 (28.57)	17 (28.81)	17 (28.33)	
**Lymphatic invasion**				0.6883
Yes	84 (70.59)	43 (72.88)	41 (68.33)	
No	35 (29.41)	16 (27.12)	19 (31.67)	
**Nerve invasion**				0.0877
Yes	76 (63.87)	33 (55.93)	43 (71.67)	
No	43 (36.13)	26 (44.07)	17 (28.33)	
**Ki-67 (%)**				0.3689
>30	94 (78.99)	49 (83.05)	45 (75.00)	
≤30	25 (21.01)	10 (16.95)	15 (25.00)	

Line 21 of Table 1: The correct percentage of low group is “10.00”.

Line 25 of Table 1: The correct percentage of low group is “36.67”.

Line 30 of Table 1: The correct percentage of low group is “20.00”.

Line 44 of Table 1: The correct content is “>30”.

The original article has been corrected.

