# Supplementary material for: Identification of tRF-29-79MP9P9NH525 as a biomarker and tumor suppressor of gastric cancer via regulating KIF14/AKT pathway
Source: Cell Death Discov. 2025 May 15;11:238. doi: 10.1038/s41420-025-02514-9 (PMC12081660; doi:10.1038/s41420-025-02514-9)
Supplement: Supplementary file 1 — Supplementary data [file 41420_2025_2514_MOESM1_ESM.pdf]

Supplemental data

Supplemental Table 1. The sequences of primers, mimics and inhibitors

Name	Sequence (5′→3′)
U6 snRNA	RT-primer: GCAGACGAGGGTACCTCCTCTCTTCTCTACTCGTGTCCTACCCTCGTCTGCAAAATA<br>Forward: GGCAGCACATATACTAAAATTGGAA<br>Reverse: CGAATTTGCGTGTCATCCTTGCG
tRF-29-79MP9P9NH525	RT-primer: GCAGAGACGGGGTACCTCCTCTCTTCTCTACTCGTGTCCTACCCCGTCTCTGCGAACGT<br>Forward: GGTACCTCCTCTCTTCTCTACT<br>Reverse: GGTGTTTCCGTAGTGTAGTGGT
NC mimics	UUGUACUACACAAAAGUACUG
NC inhibitor	CAGUACUUUUGUGUAGUACAA
tRF-29-79MP9P9NH525 mimics	GUUUCCGUAGUGUAGUGGUUAUCACGUUC
tRF-29-79MP9P9NH525 inhibitor	GAACGUGAUAACCACUACACUACGGAAAC
KIF14-F	GTGGAAGGGGAAGAACACGA
KIF14-R	CAGCGGGACTAATCGTAGCA
AQP11-F	GCTGACGCTCGTCTACTTCT
AQP11-R	TGAGCAAGTCGACTCGGATG
S1PR3-F	GATCCTCTACGCACGCATCT
S1PR3-R	CACAGCCAACACGATGAACC
THBS1-F	GCAAAGAAGTGCCTGATGCC
THBS1-R	CGTTCTTGTTGCAGTCGTGG
GAPDH-F	AAGGTGAAGGTCGGAGTCAA
GAPDH-R	AATGAAGGGGTCATTGATGG
si-NC (Sense)	UUCUCCGAACGUGUCACGUTT
si-NC (Antisense)	ACGUGACACGUUCGGAGAATT
si-KIF14 -1 (Sense)	GCUGCAUUUGAAGUCGGAUAU
si-KIF14 -1 (Antisense)	AUAUCCGACUUCAAAUGCAGC
si-KIF14 -2 (Sense)	CGGCAAGAAAUAACAUCCUUA
si-KIF14 -2 (Antisense)	UAAGGAUGUUAUUUCUUGCCG
si-KIF14 -3 (Sense)	GCUGCAUUUGAAGUCGGAUAU
si-KIF14 -3 (Antisense)	AUAUCCGACUUCAAAUGCAGC

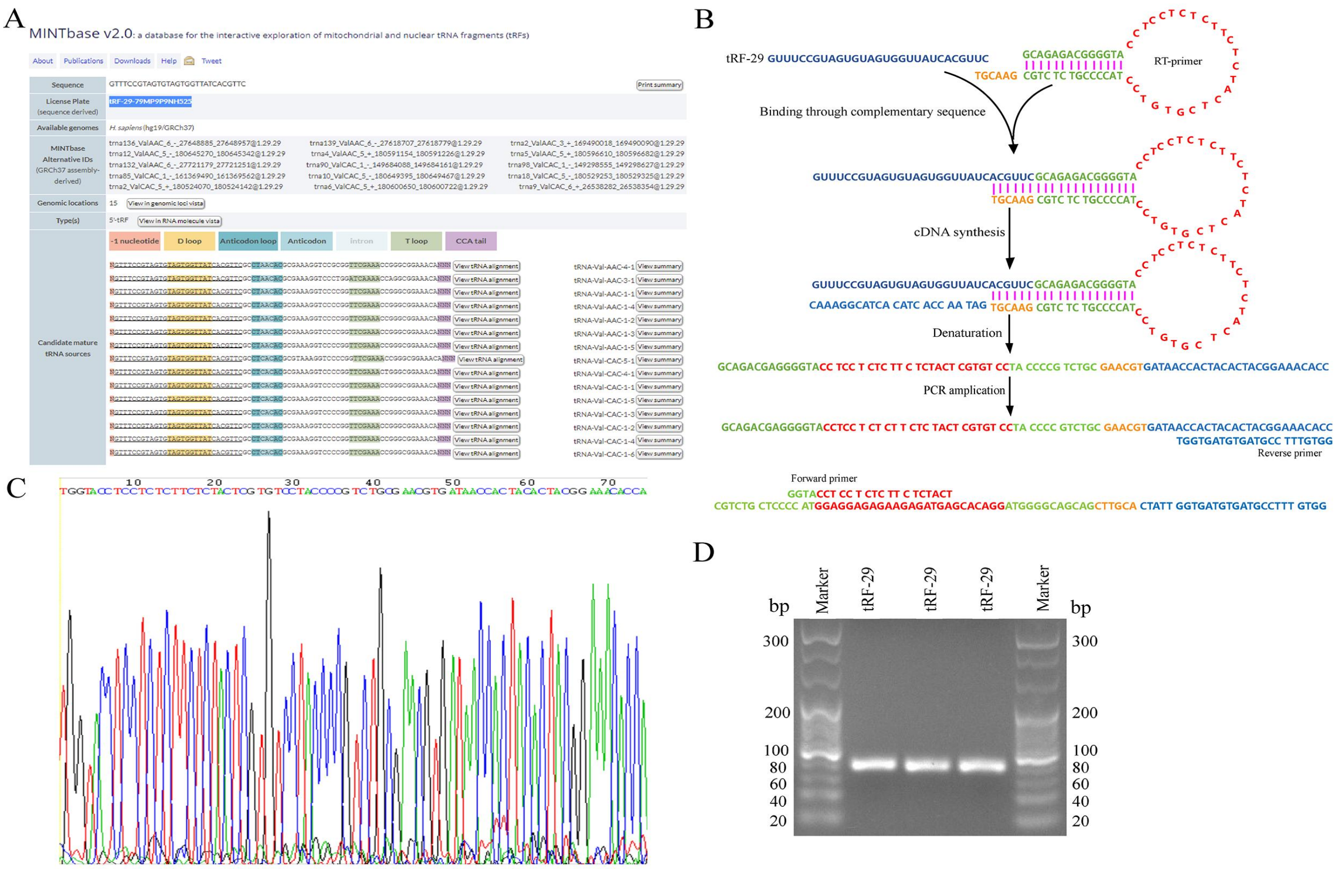

Supplemental Figure 1. Establishment of tRF-29-79MP9P9NH525 (tRF-29) detection system. (A) tRF-29 is a type of 5′-tRF that derived from tRNA-Val-AAC. (B) The sequences of primers for reverse reaction and amplification, and the process of qRT-PCR. (C) The T-A cloning sequencing result of tRF-29 qRT-PCR product. (D) Agarose gel electrophoresis results tRF-29 qRT-PCR products of three tissue samples.

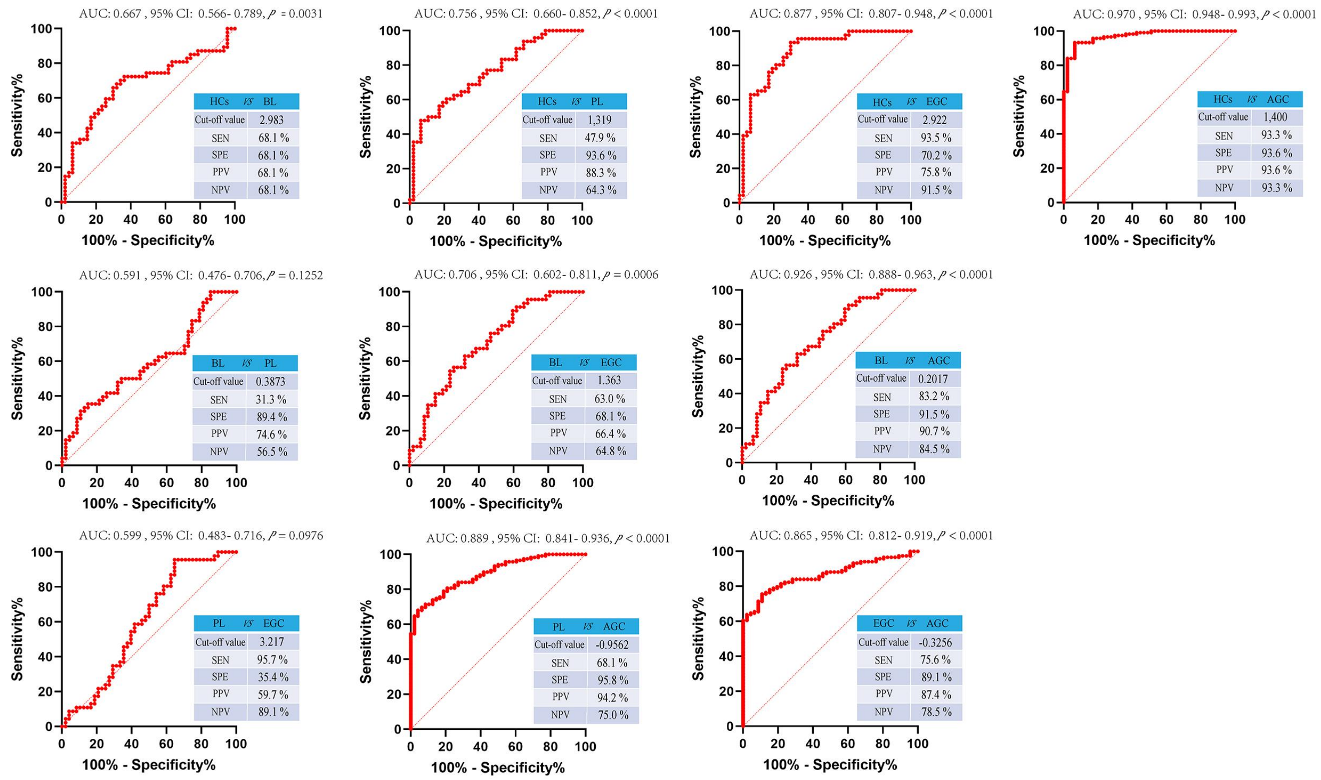

Supplemental Figure 2. The value of tRF-29-79MP9P9NH525 (tRF-29) in monitoring different gastric mucosa. tRF-29 shows discriminating efficiency in different gastric mucosa. HCs, healthy controls (*n*=47); BL, benign lesion (*n*=47); PL, precancerous lesion (*n*=48); EGC, early gastric cancer (*n*=46); AGC, advanced gastric cancer (*n*=119); SEN, sensitivity; SPE, specificity; PPV, positive predictive value; NPV, negative predictive value.

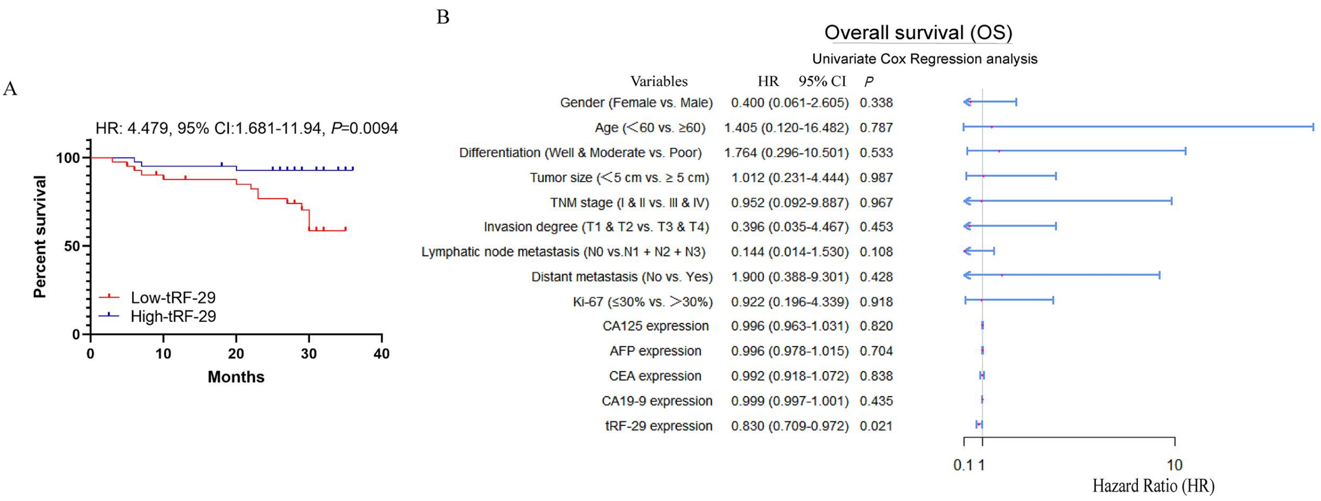

Supplemental Figure 3. The prognostic value of tRF-29-79MP9P9NH525 (tRF-29) in gastric cancer (GC). (A) Low level of tRF-29 was related with poor overall survival (*n*=84). (B) Multivariate Cox analysis validated the predictive value of tRF-29 in GC.

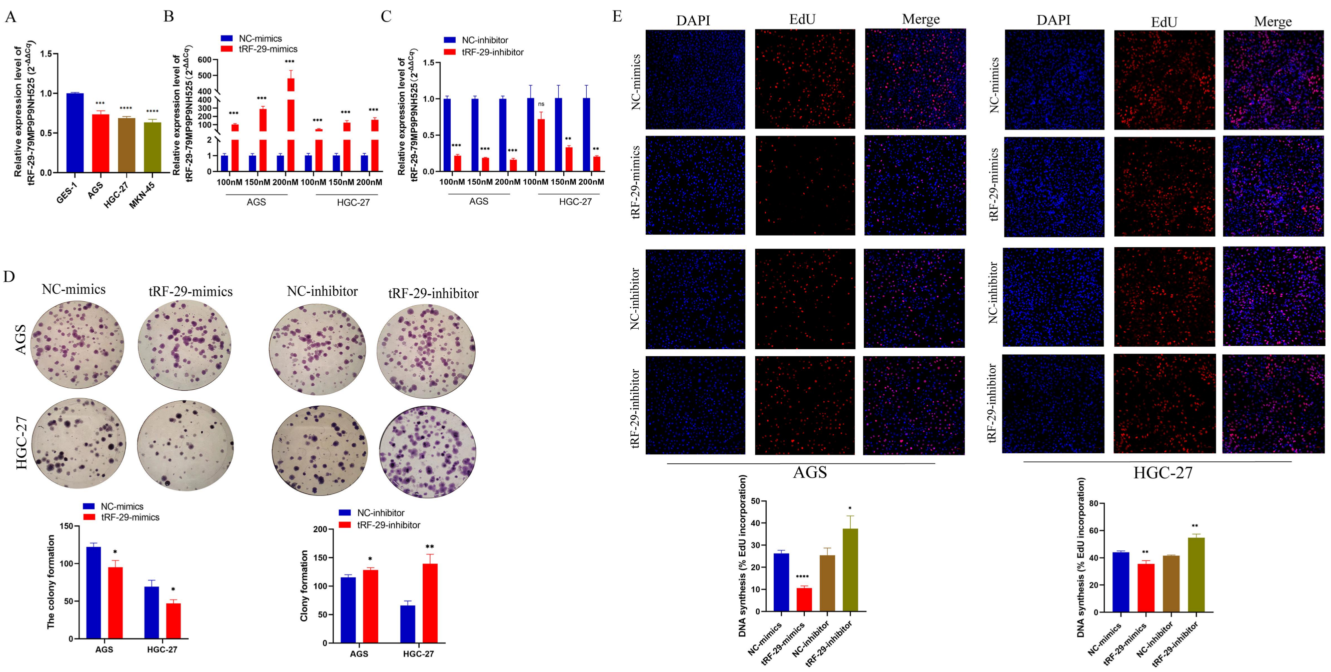

Supplemental Figure 4. The effects of tRF-29-79MP9P9NH525 (tRF-29) on the proliferation of gastric cancer. (A) tRF-29 is downregulated in AGS, HGC-27, and MKN-45 cells compared with GES-1 cells. (B, C) The transfection efficiency of tRF-29-mimics (B) and tRF-29-inhibitor (C) was quantified by qRT-PCR. (D) The effects of tRF-29 on the growth of gastric cancer cells. (E) The effects of tRF-29 on the proliferative activity were determined by EdU assay. *n*=3; **P*<0.05, ***P*<0.01, *****P*<0.0001.

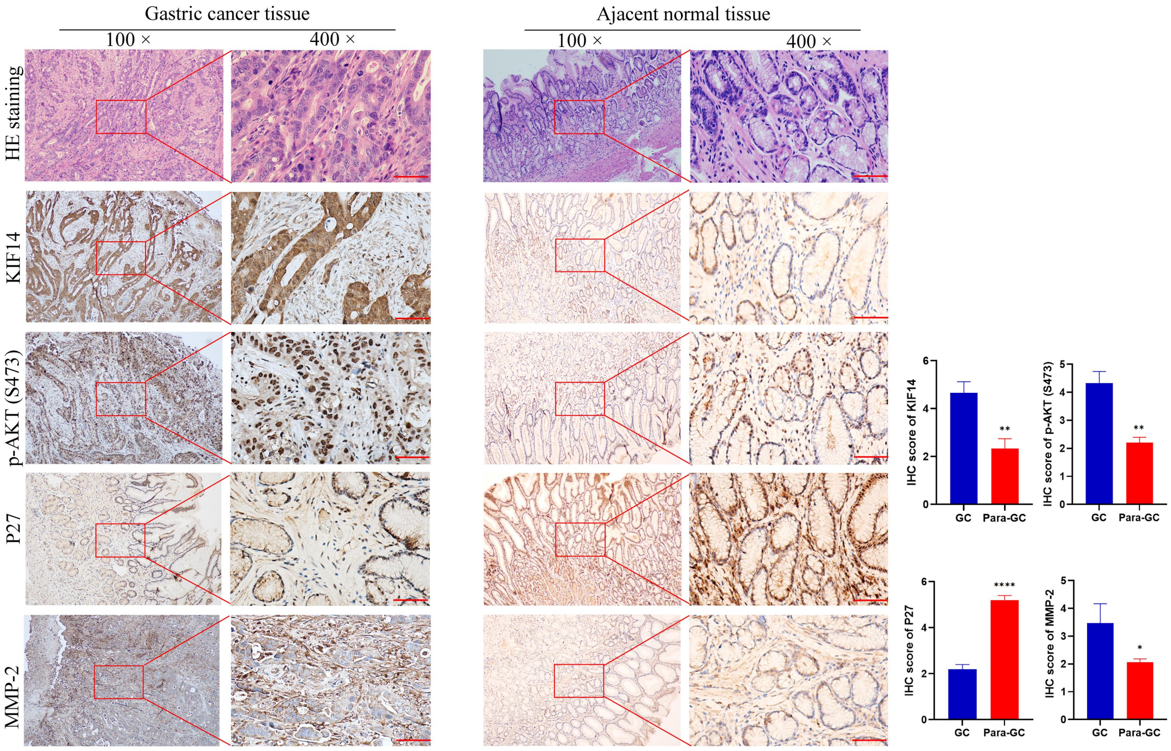

Supplemental Figure 5. Differential tRF-29 level shows various effects on KIF14/AKT pathway in human tissue samples. Immunohistochemistry (IHC) staining of the protein levels of KIF14, P27, MMP-2, and p-AKT(S473) in human tissue samples. *n*=3; *P<0.05, **P<0.01, ****P<0.0001.

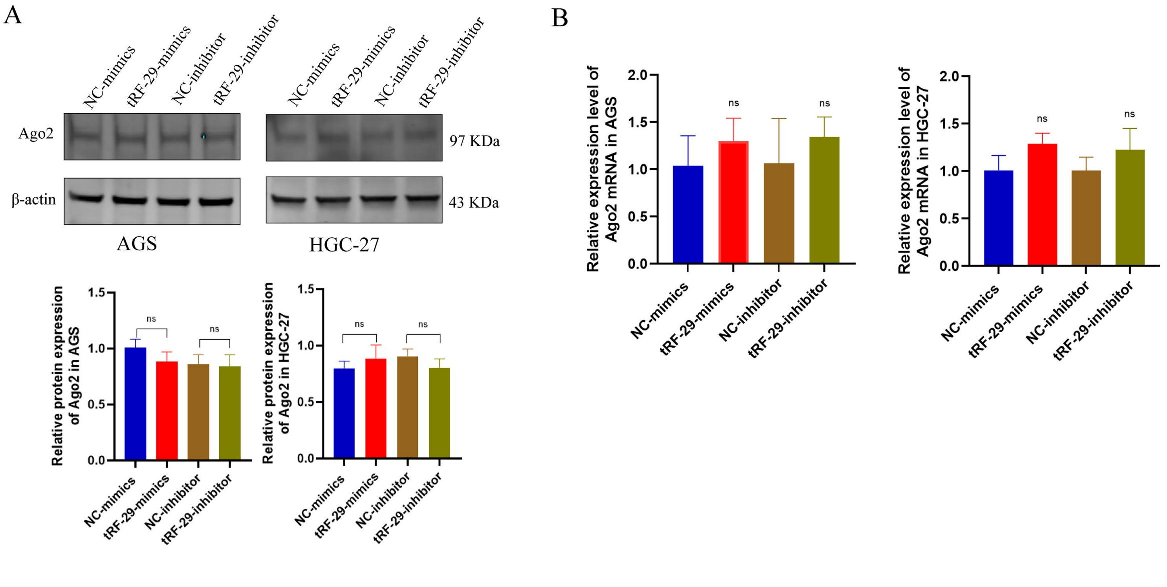

Supplemental Figure 6. tRF-29 shows no effects on Ago2 expression. (A) Ago2 protein level was determined by WB in GC cells with tRF-29 upregulation or downregulation. (B) Ago2 mRNA level was determined by qRT-PCR. *n*=3; ns, no significance.

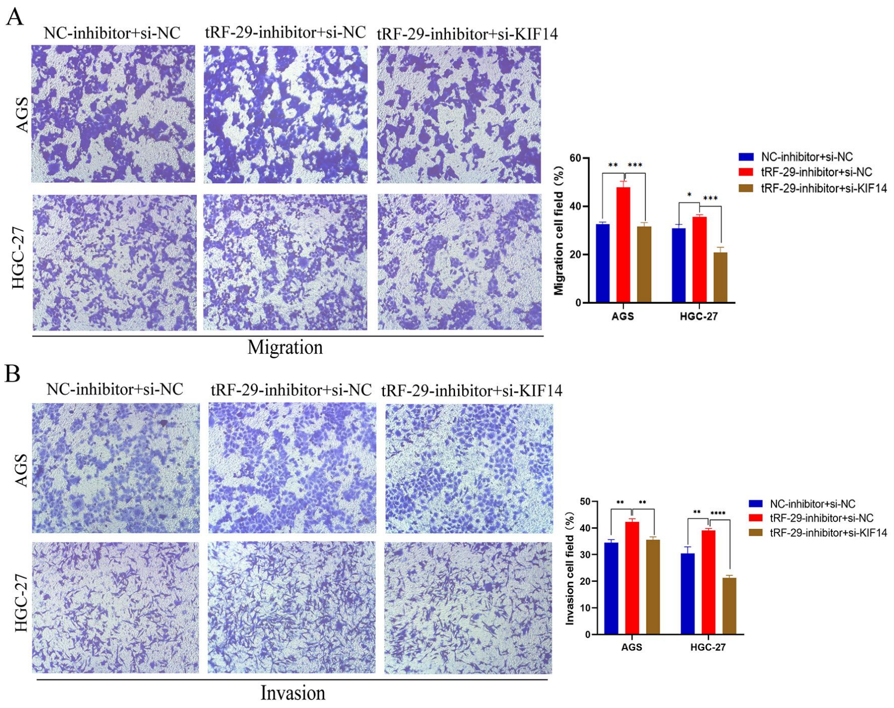

Supplemental Figure 7. siRNA of KIF14 counteracts tRF-29's impact on gastric cancer cell metastasis. Downregulation of KIF14 reverses tRF-29's effects on gastric cancer cells' migration (A) and invasion (B). n=3; *P<0.05, **P<0.01, ****P<0.0001.
