# Supplementary material for: Identification of tRF-29-79MP9P9NH525 as a biomarker and tumor suppressor of gastric cancer via regulating KIF14/AKT pathway
Source: Cell Death Discov. 2025 May 15;11:238. doi: 10.1038/s41420-025-02514-9 (PMC12081660; doi:10.1038/s41420-025-02514-9)
Supplement: Supplementary file 2 — Raw data of WB [file 41420_2025_2514_MOESM2_ESM.pptx]

## Slide 1
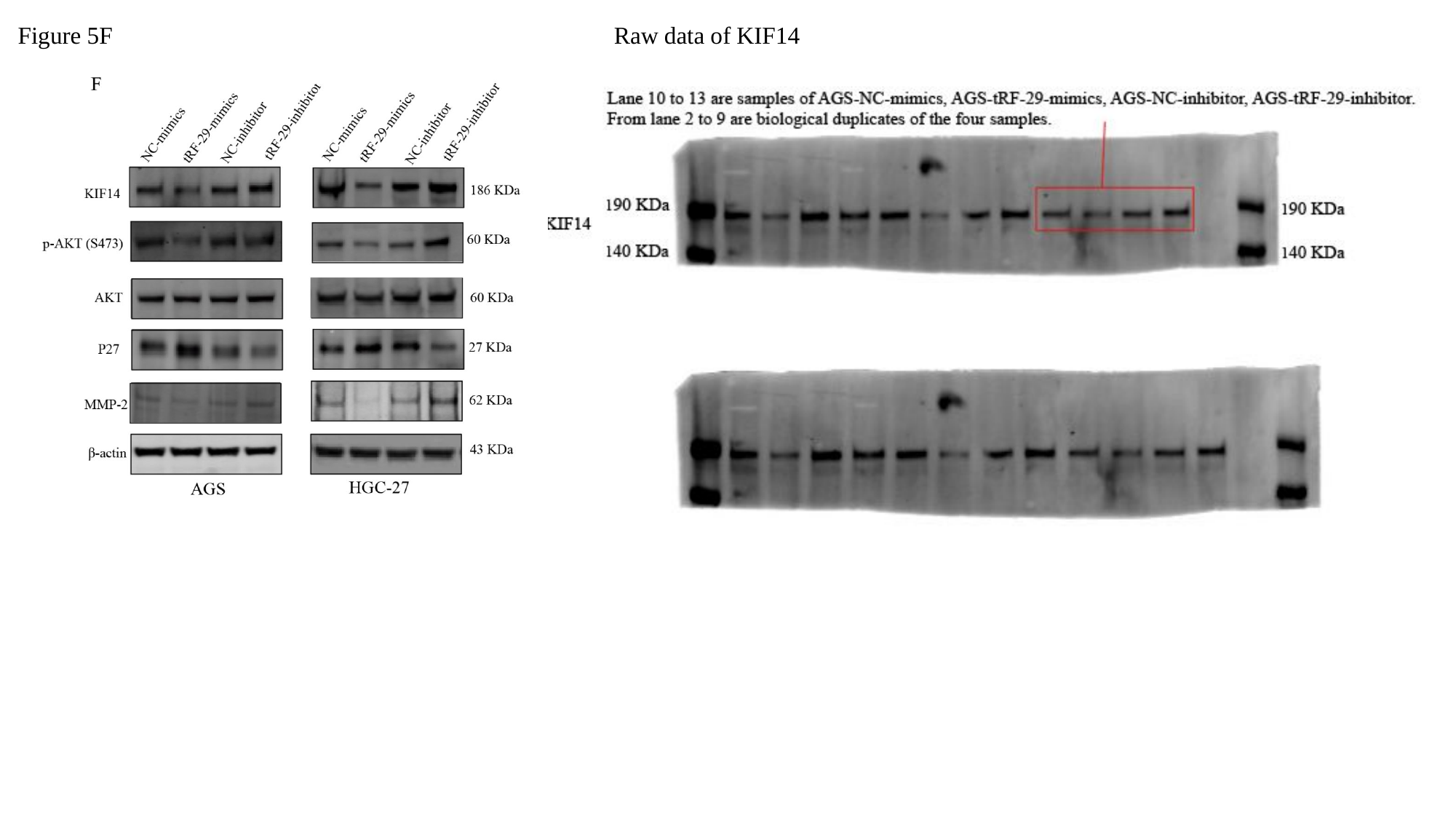

Figure 5F
Raw data of KIF14

## Slide 2
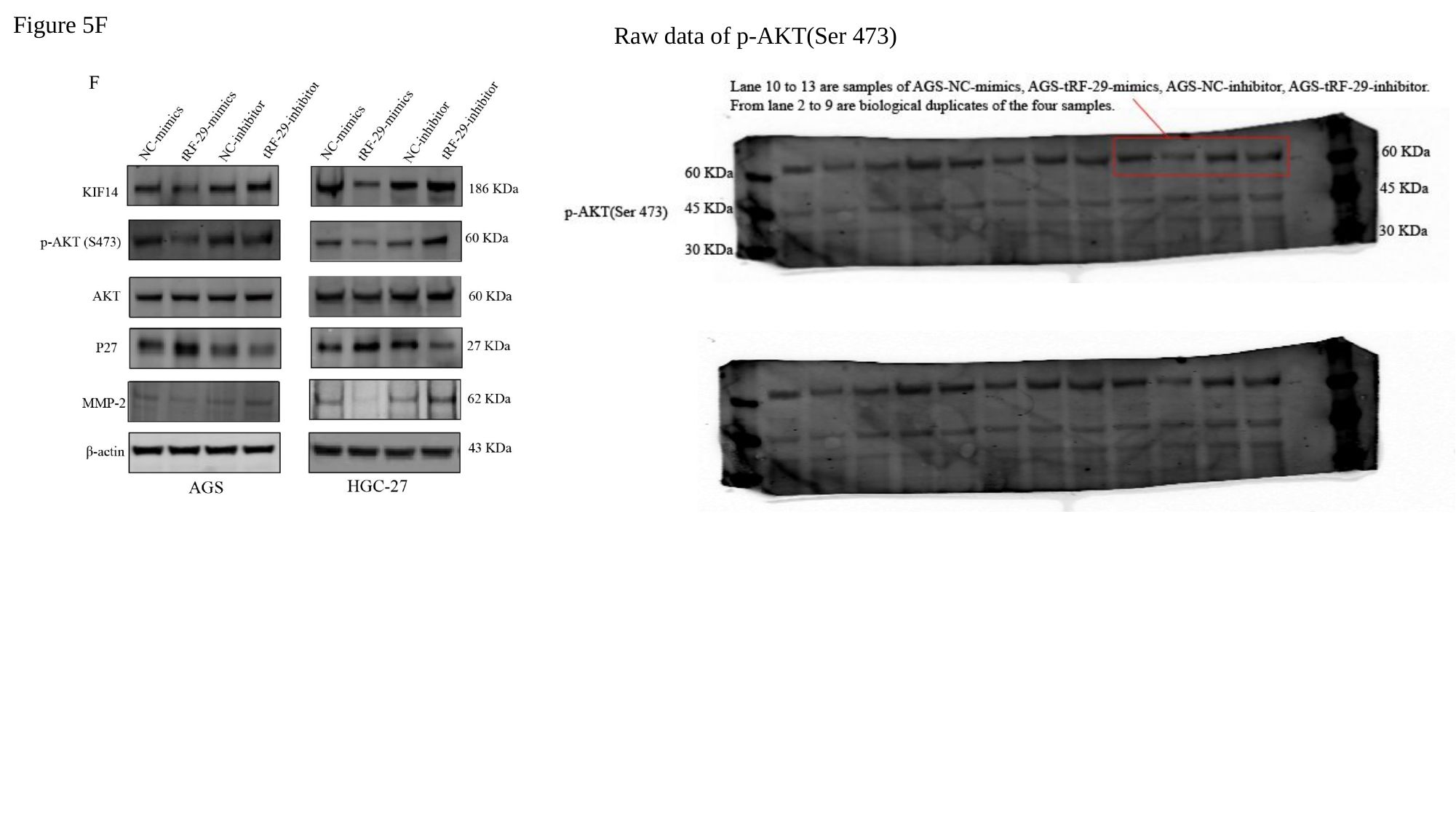

Figure 5F
Raw data of p-AKT(Ser 473)

## Slide 3
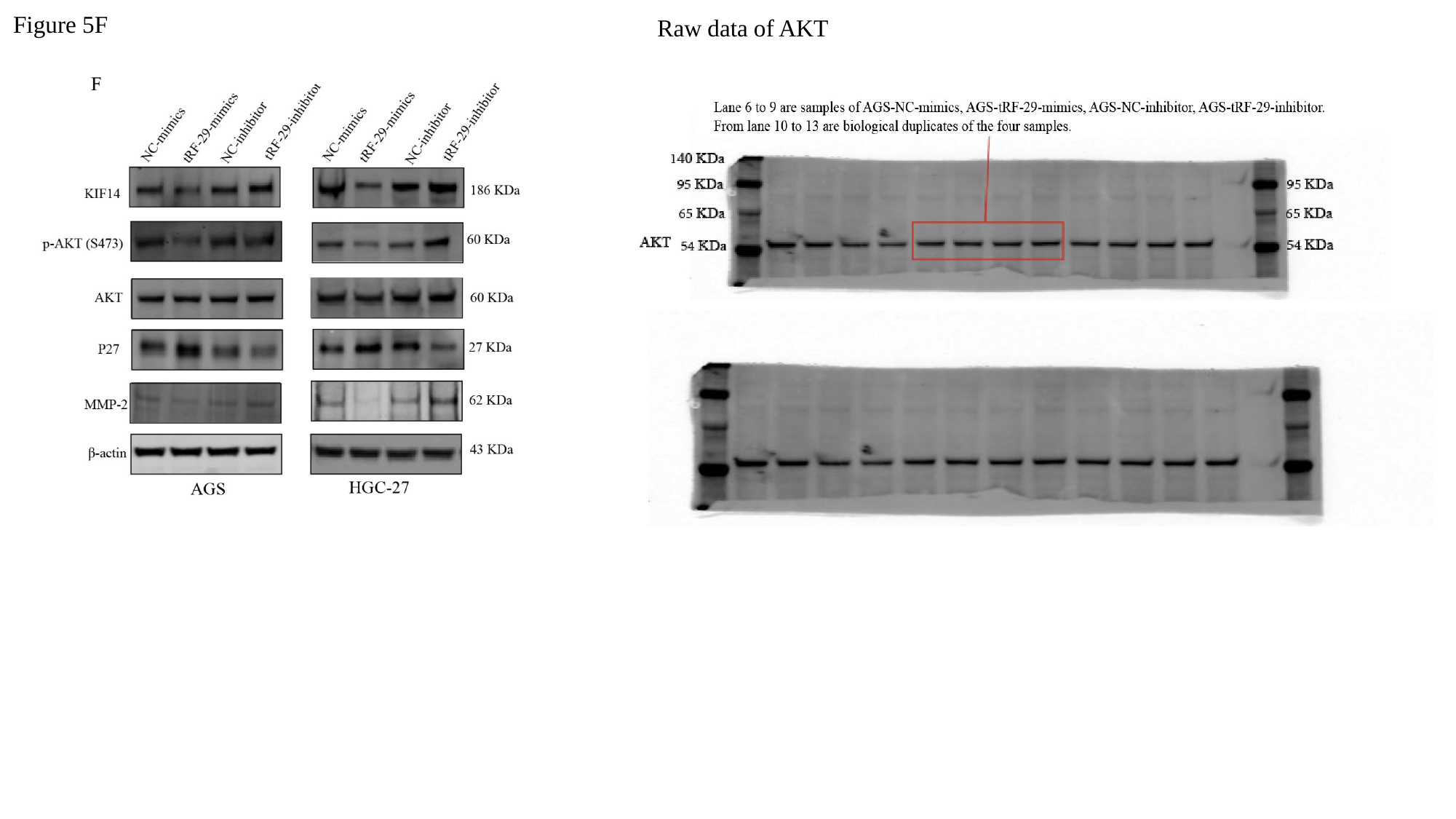

Figure 5F
Raw data of AKT

## Slide 4
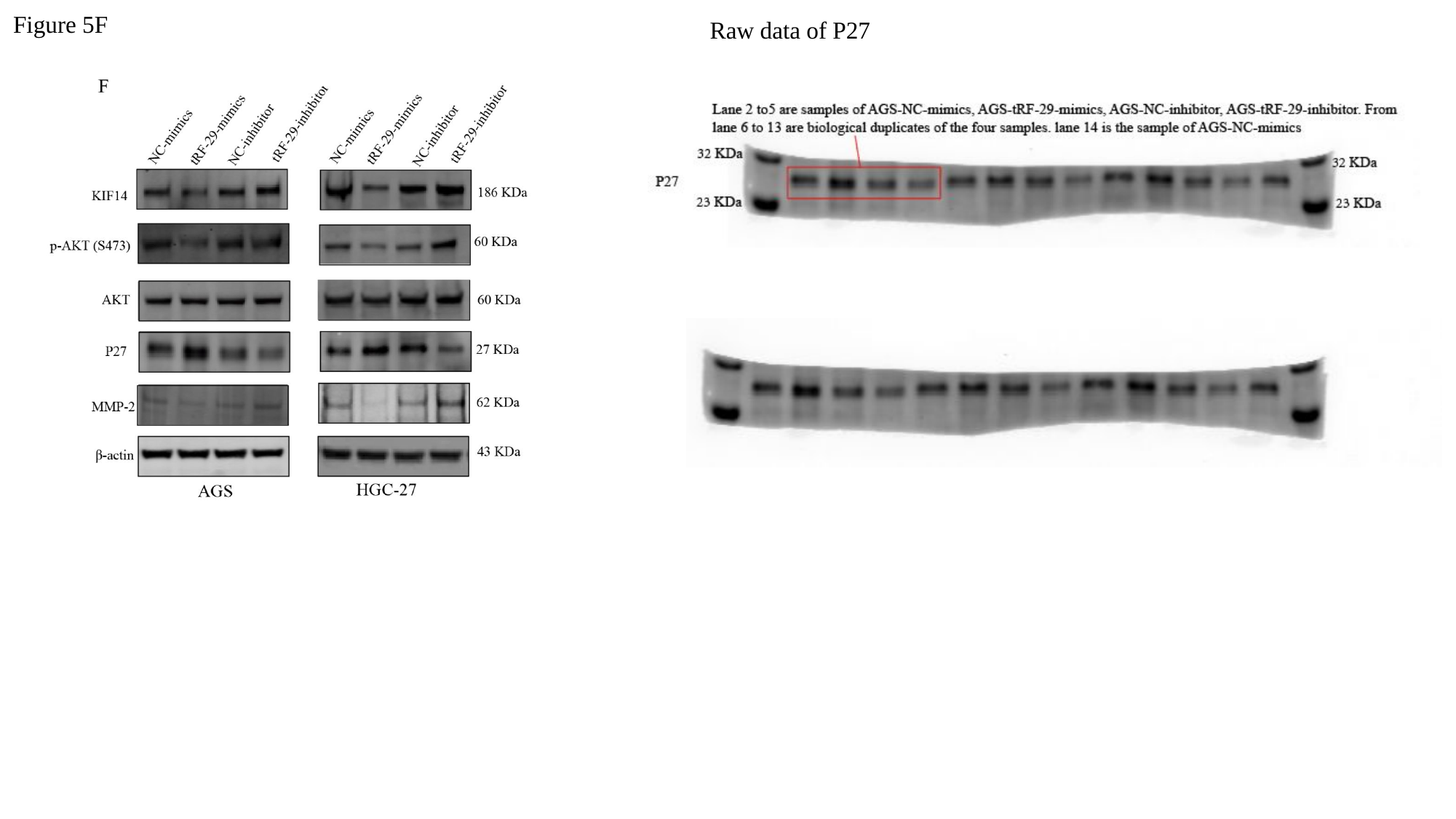

Figure 5F
Raw data of P27

## Slide 5
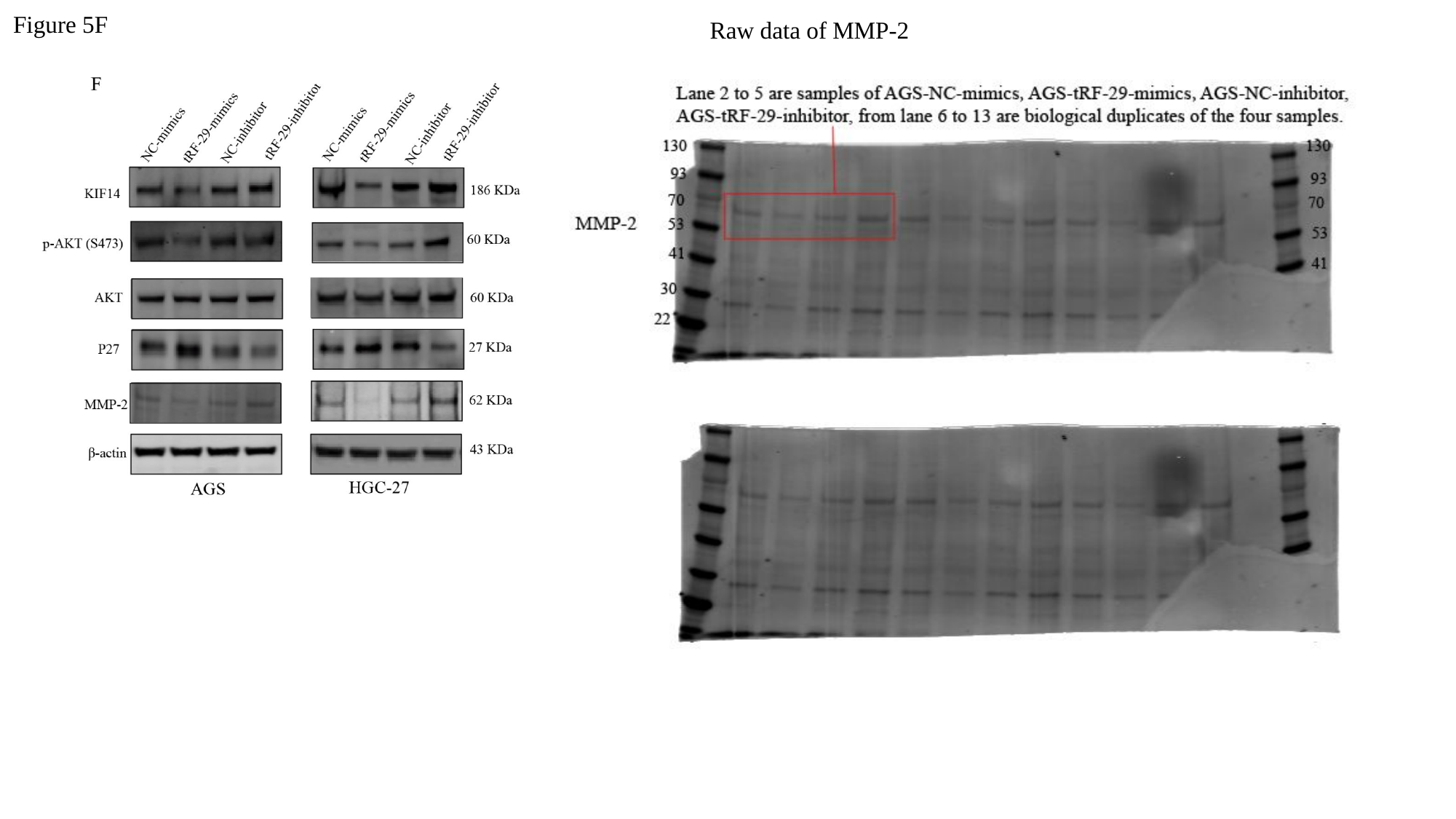

Figure 5F
Raw data of MMP-2

## Slide 6
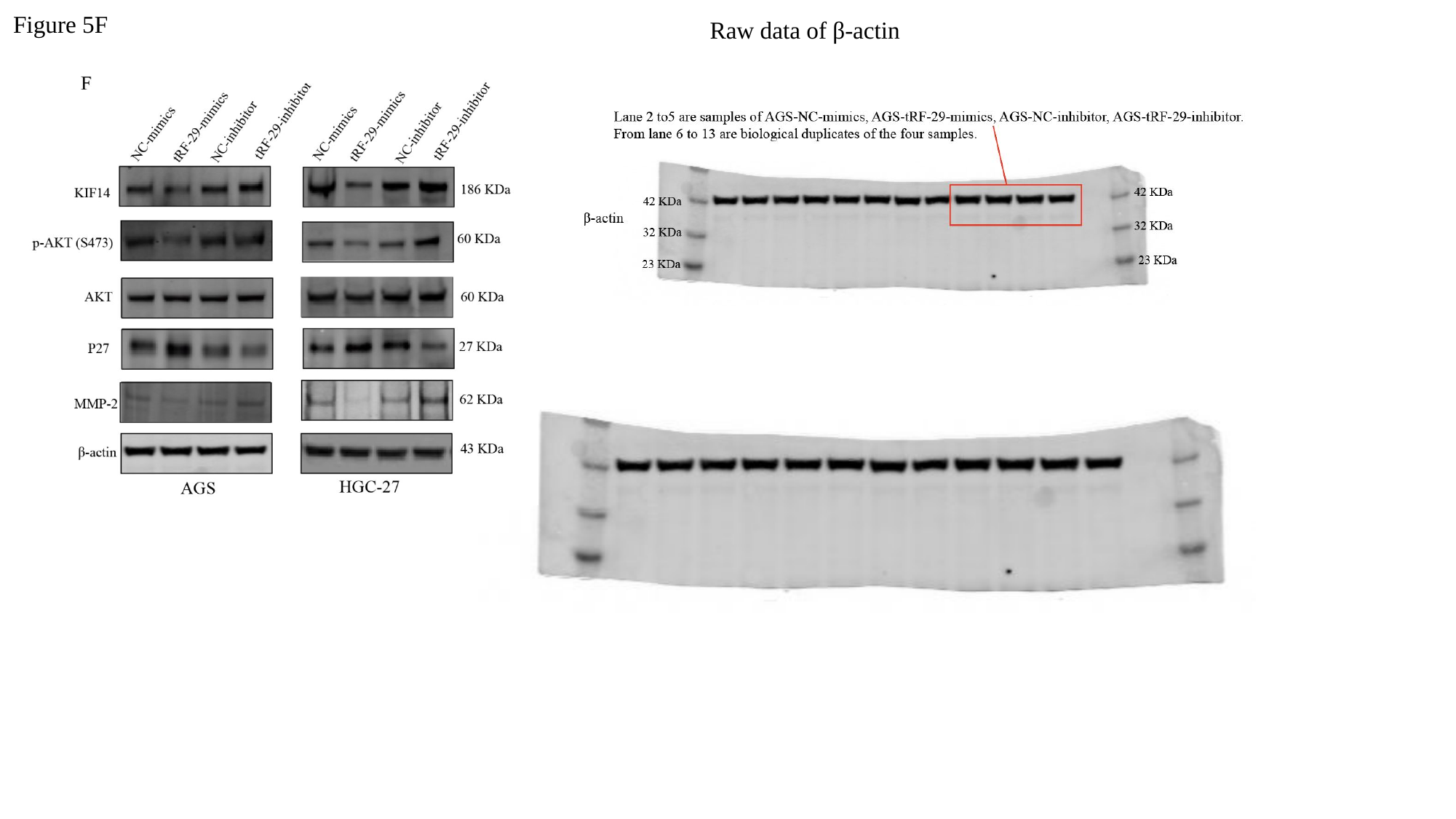

Figure 5F
Raw data of β-actin

## Slide 7
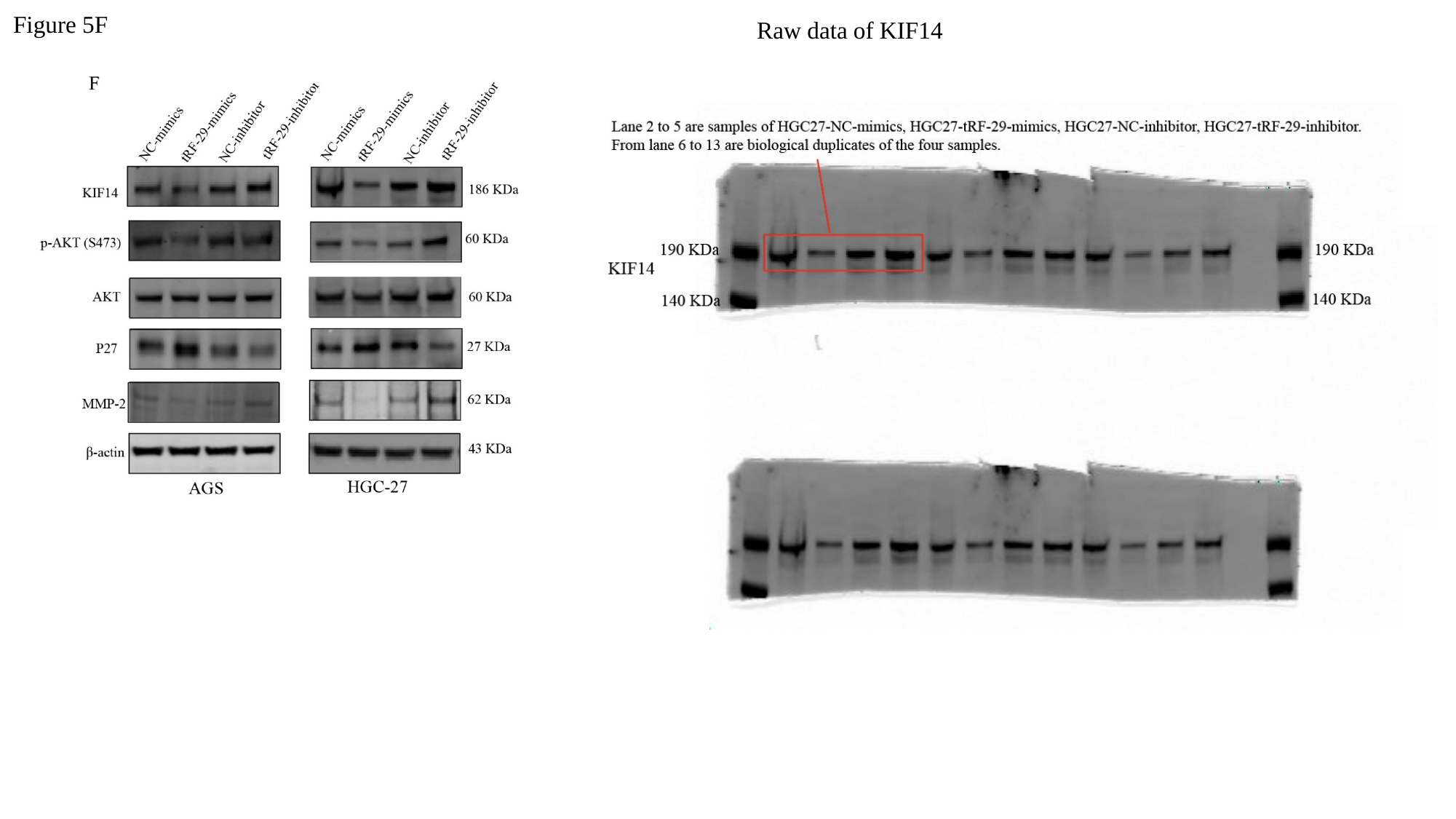

Figure 5F
Raw data of KIF14

## Slide 8
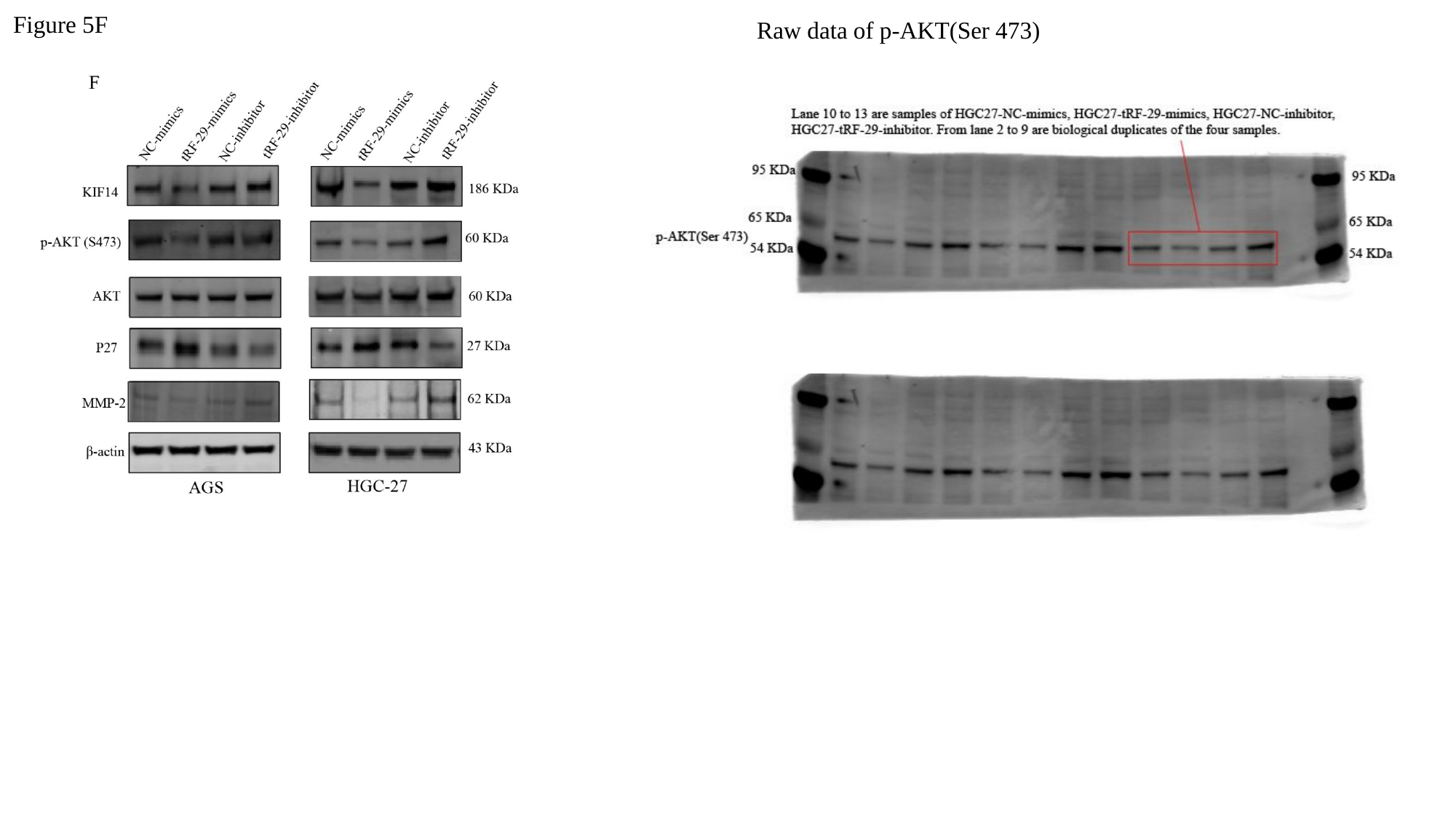

Figure 5F
Raw data of p-AKT(Ser 473)

## Slide 9
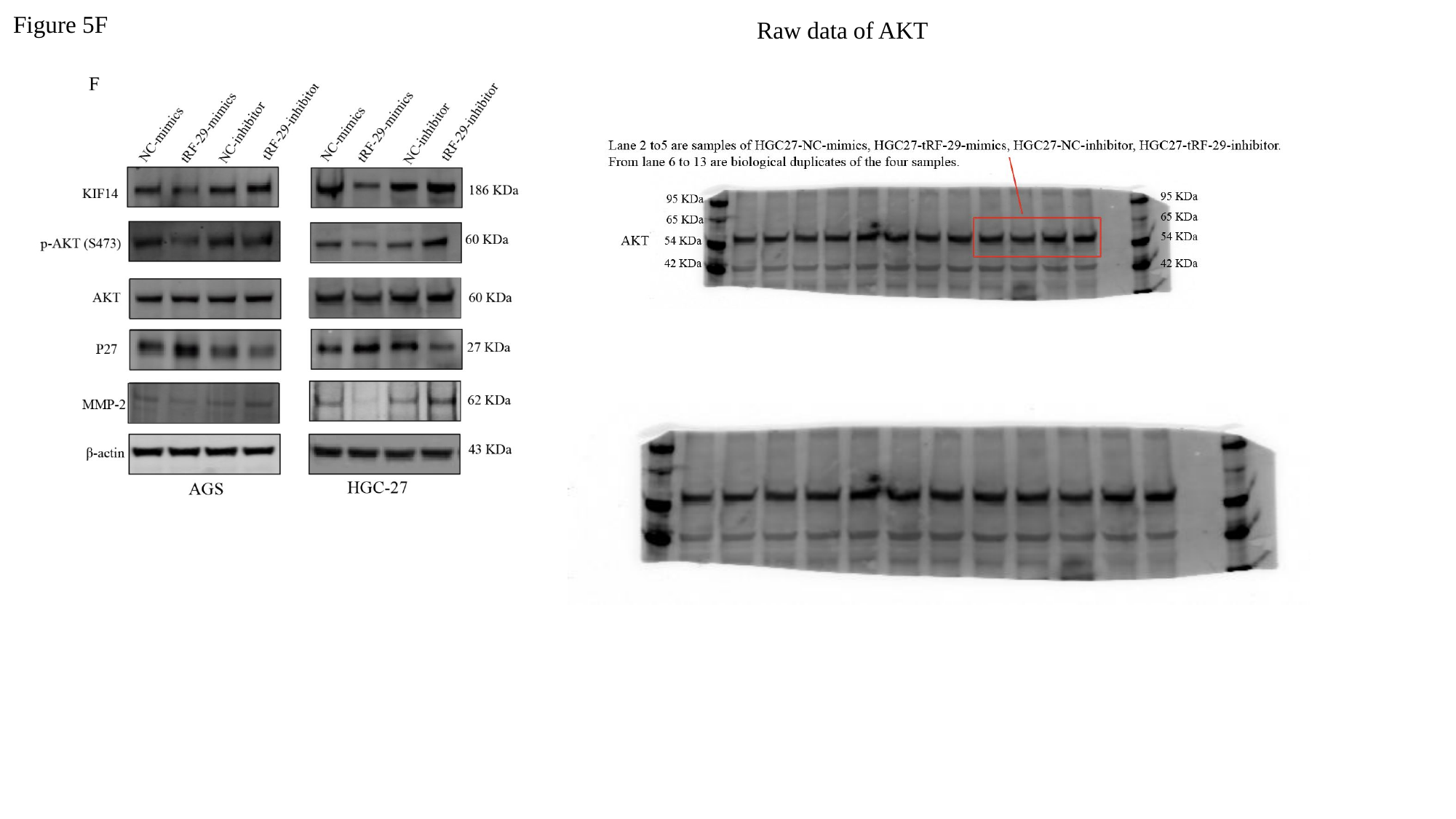

Figure 5F
Raw data of AKT

## Slide 10
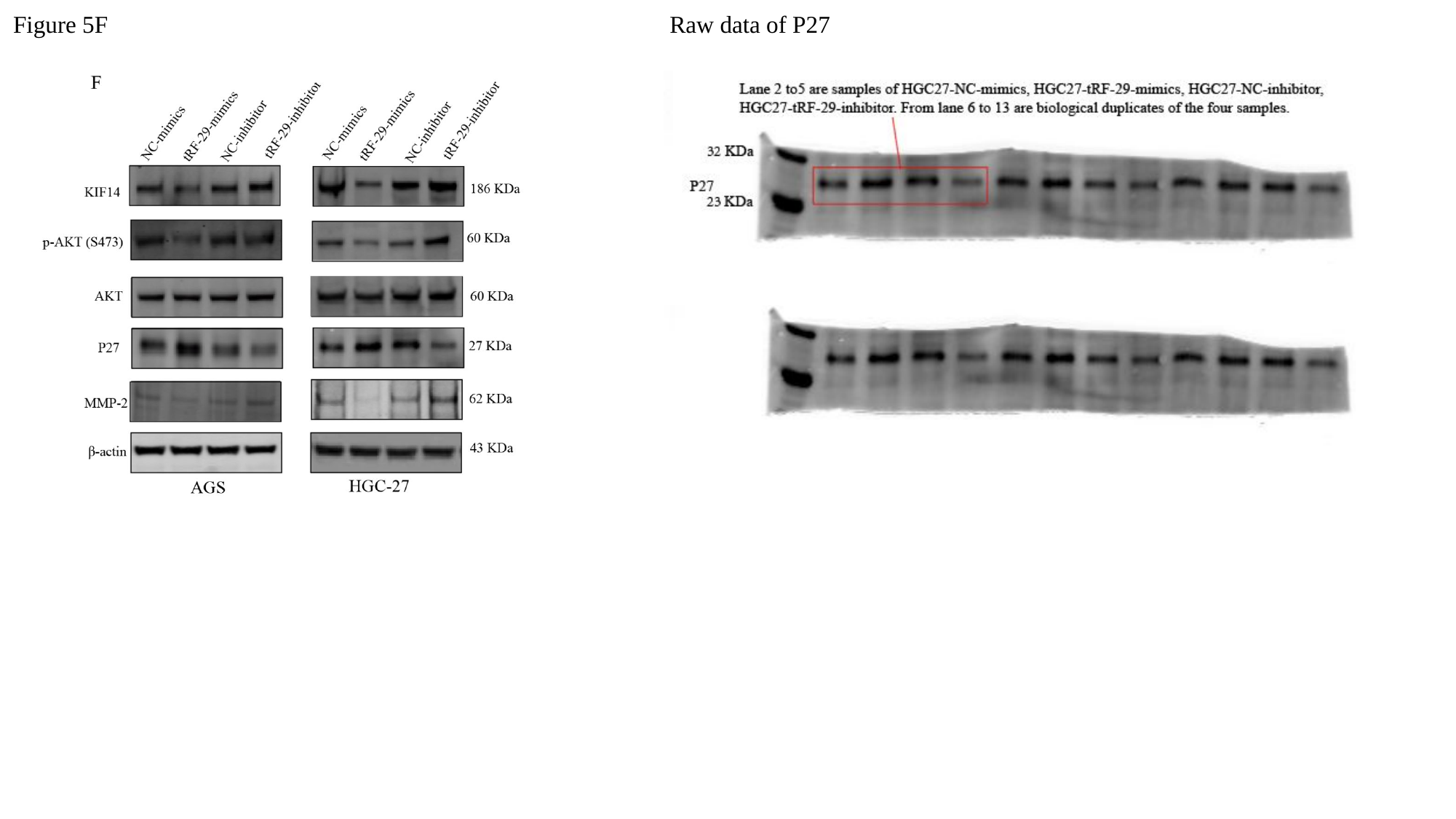

Figure 5F
Raw data of P27

## Slide 11
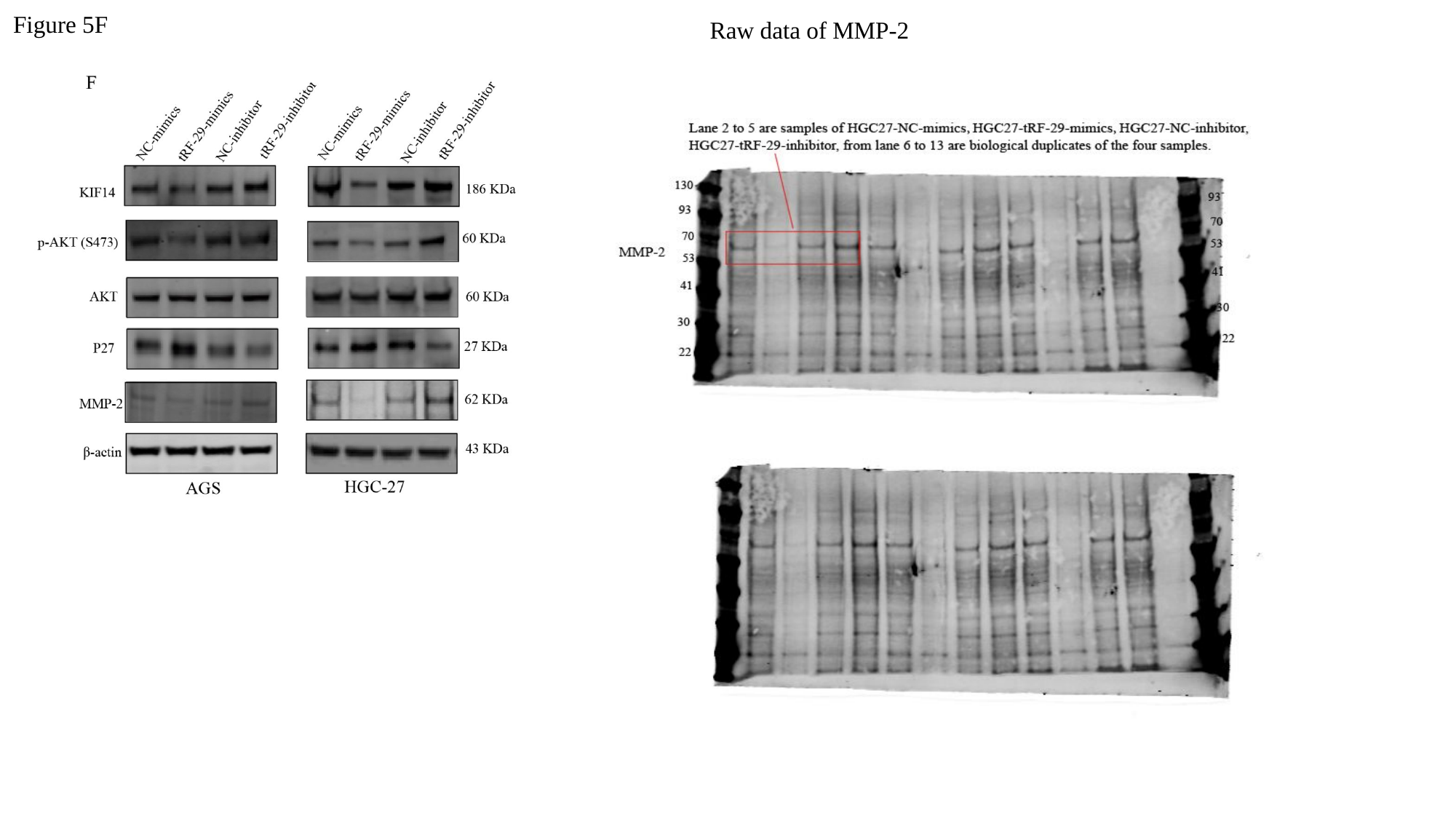

Figure 5F
Raw data of MMP-2

## Slide 12
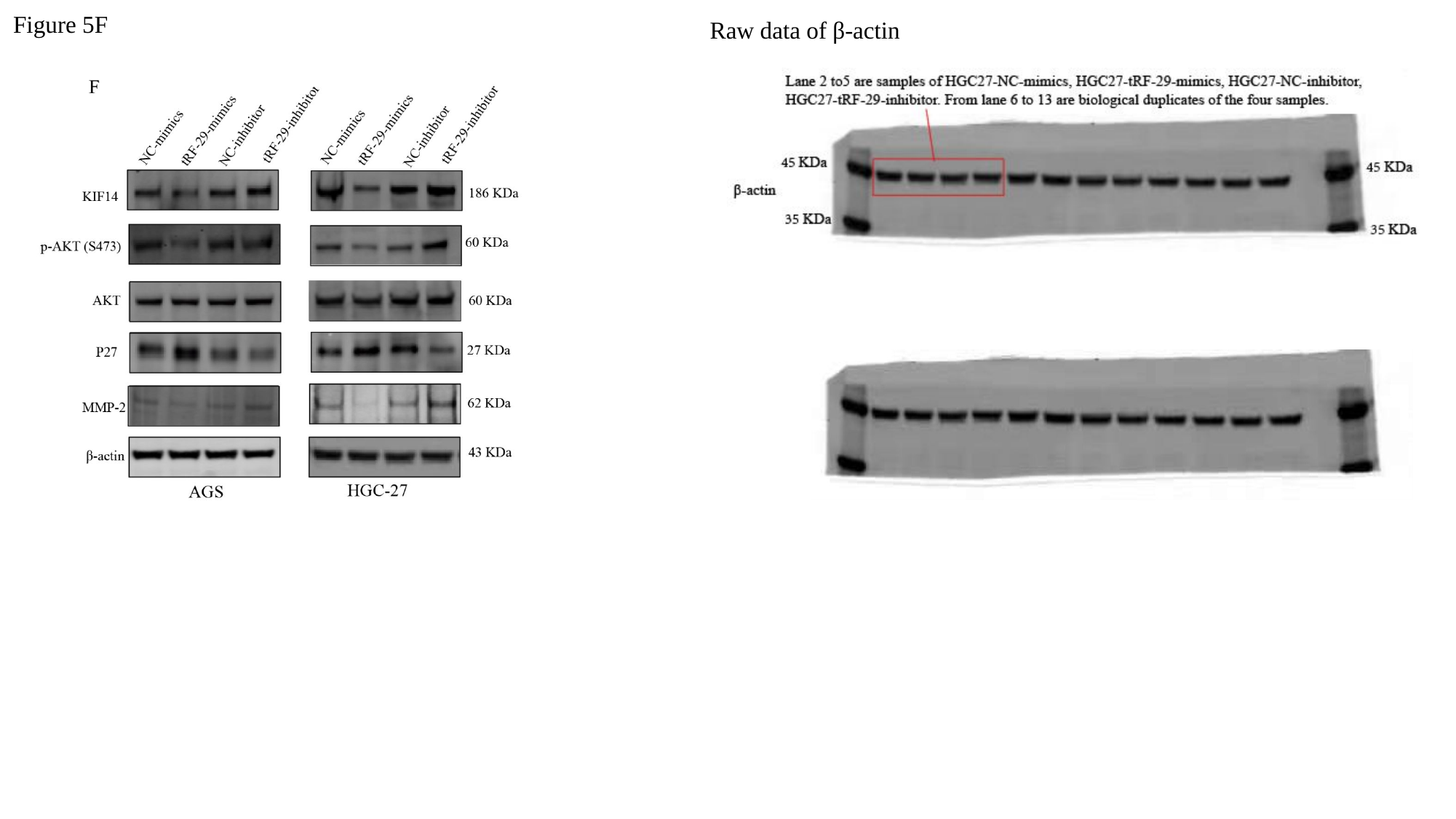

Figure 5F
Raw data of β-actin

## Slide 13
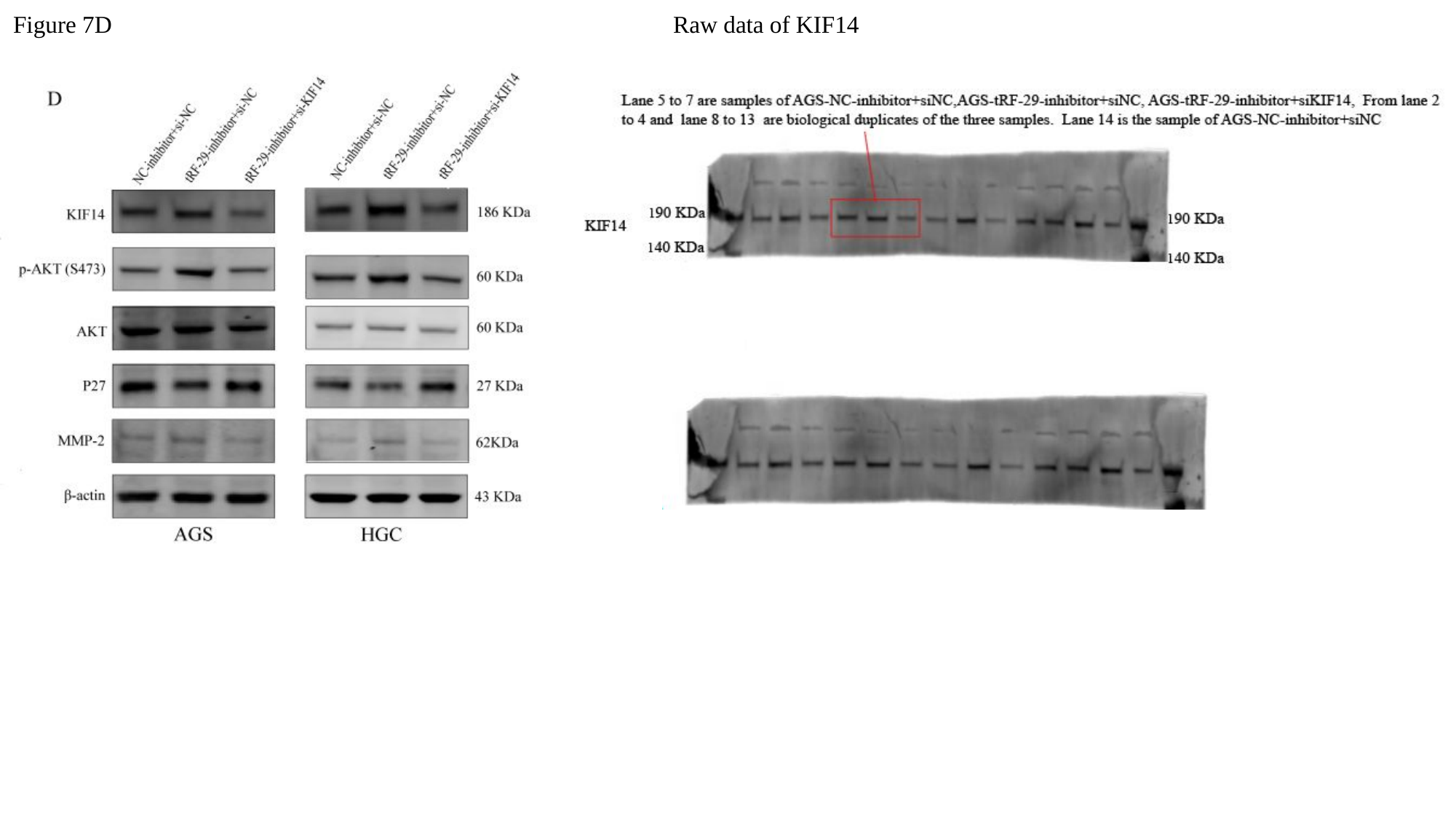

Figure 7D
Raw data of KIF14

## Slide 14
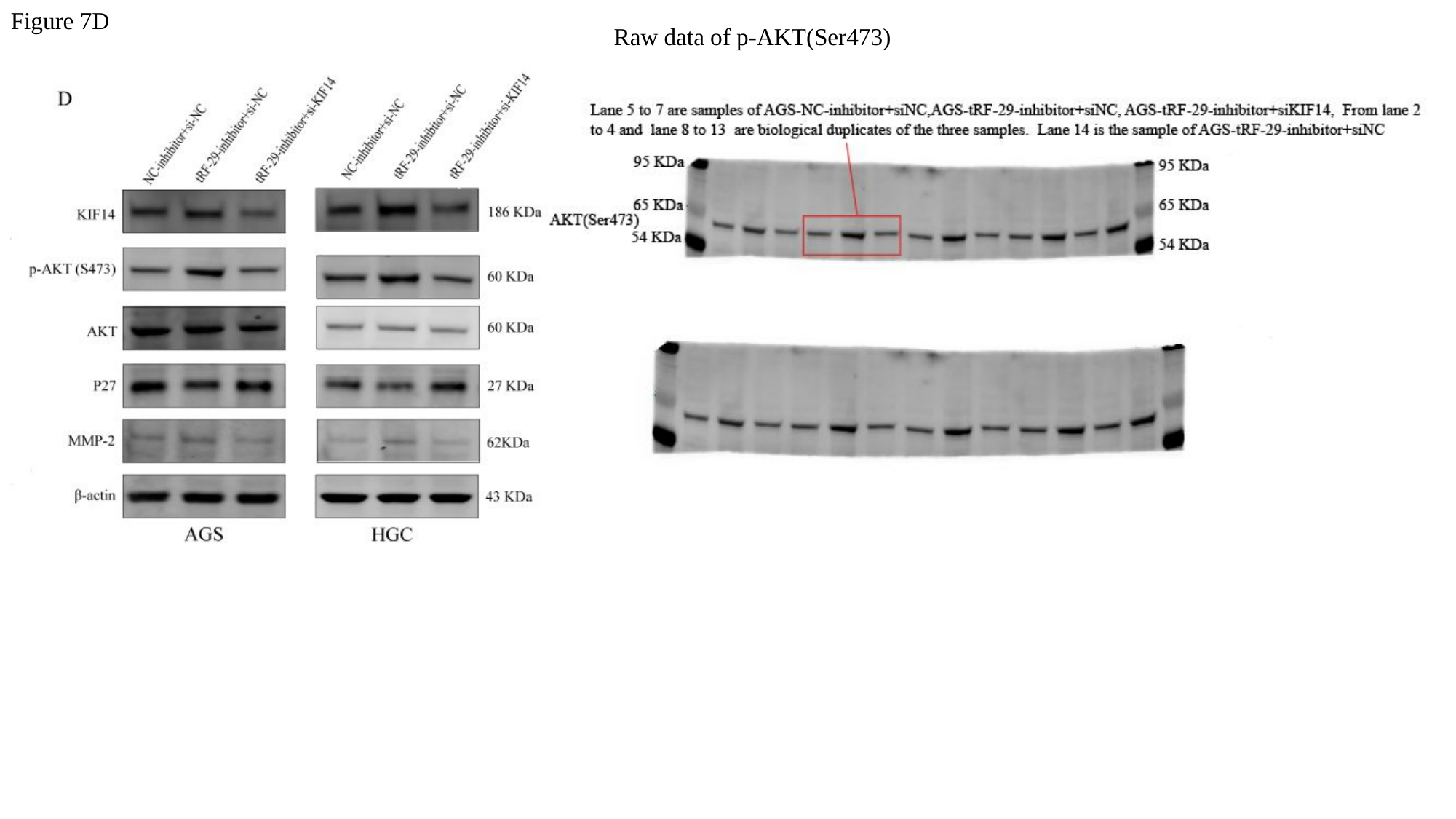

Figure 7D
Raw data of p-AKT(Ser473)

## Slide 15
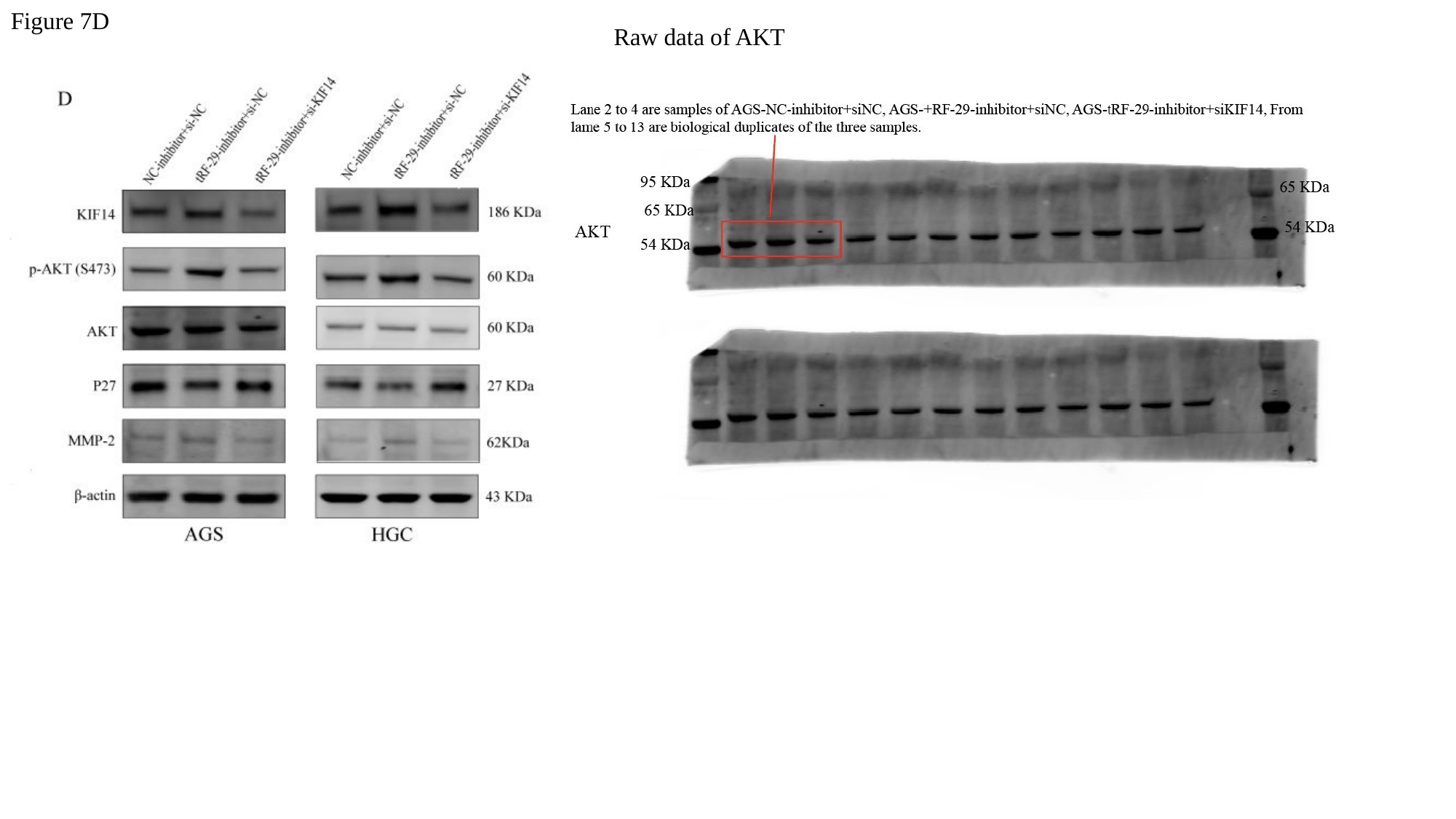

Figure 7D
Raw data of AKT

## Slide 16
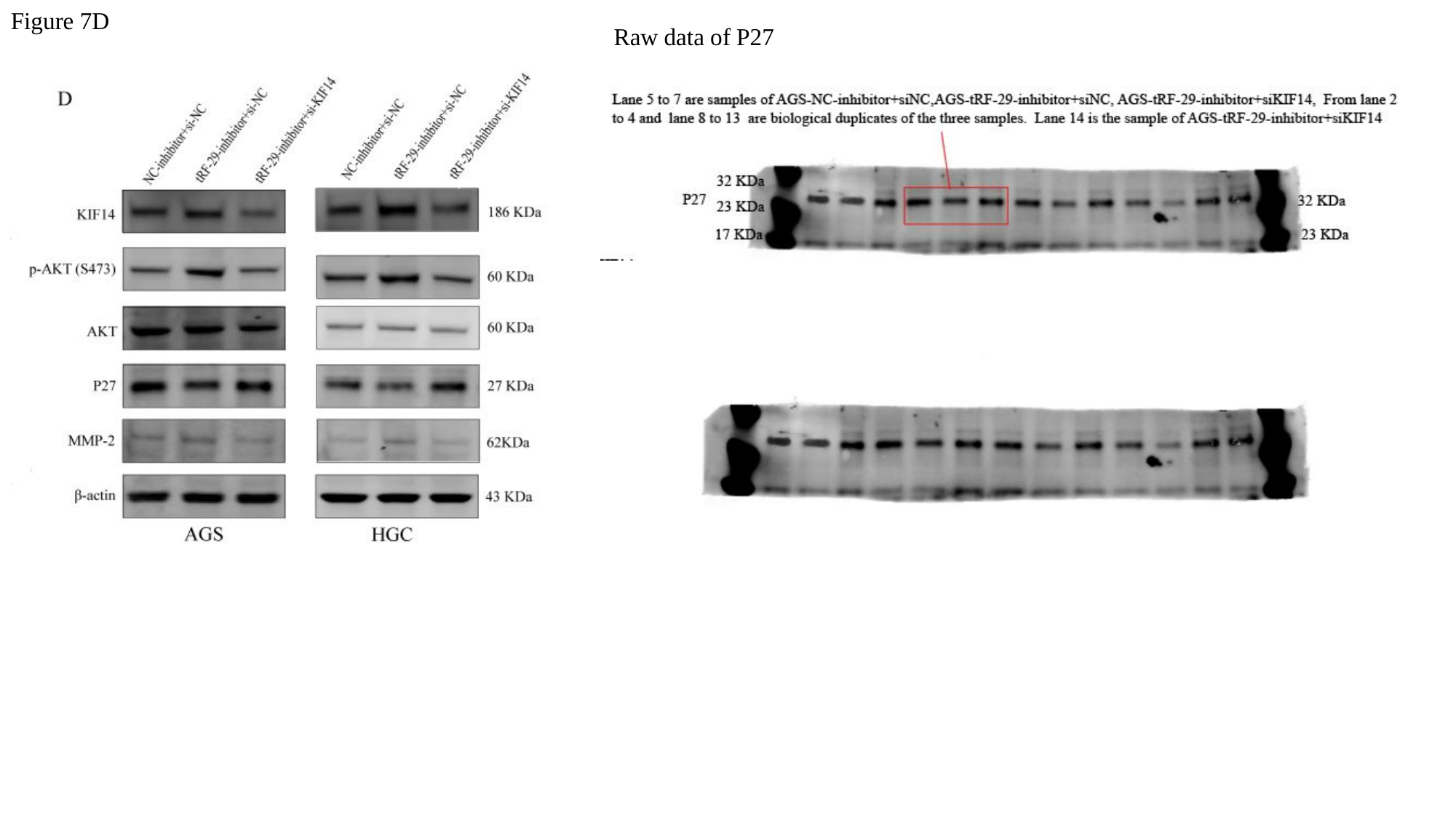

Figure 7D
Raw data of P27

## Slide 17
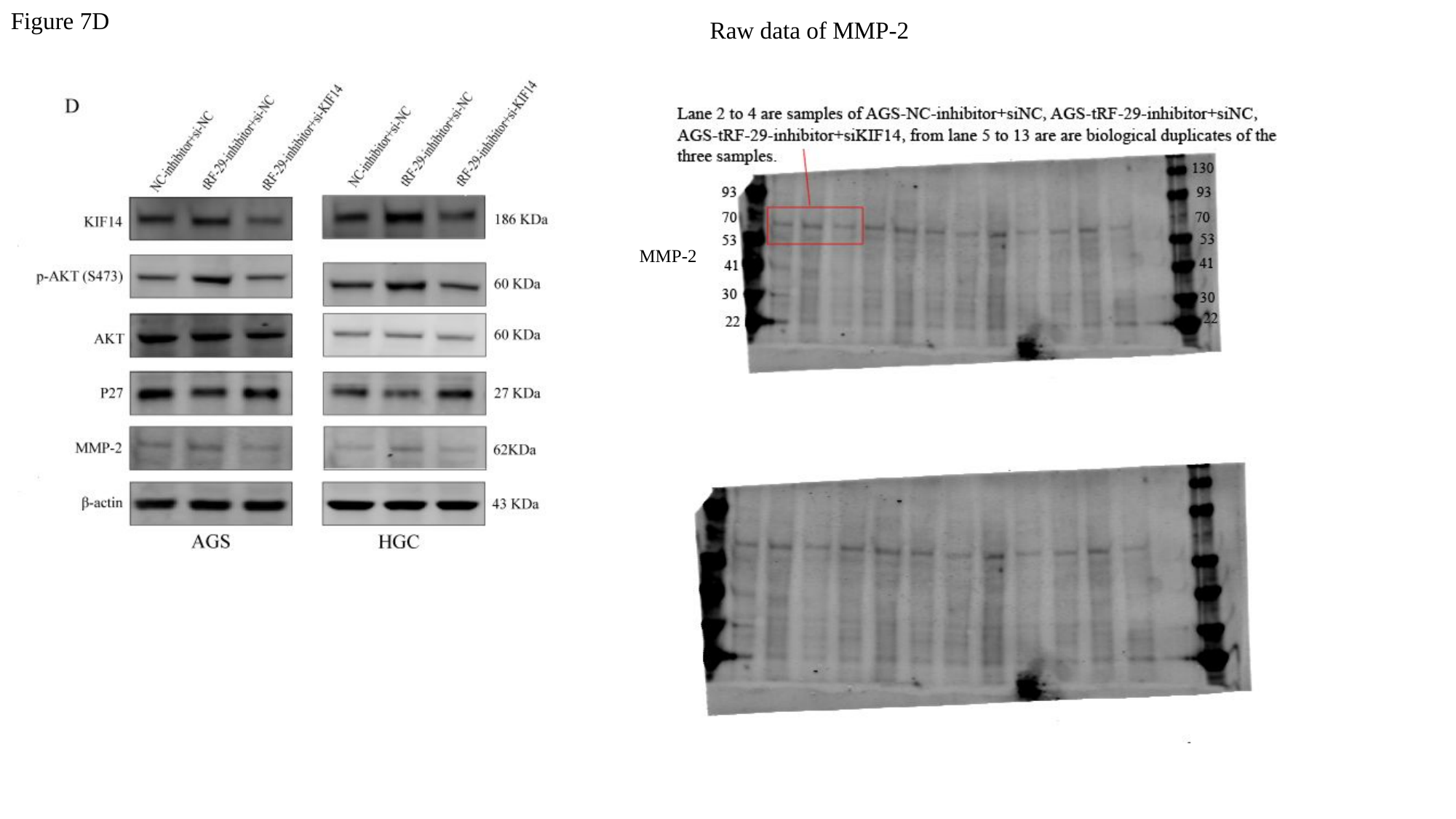

Figure 7D
Raw data of MMP-2
MMP-2

## Slide 18
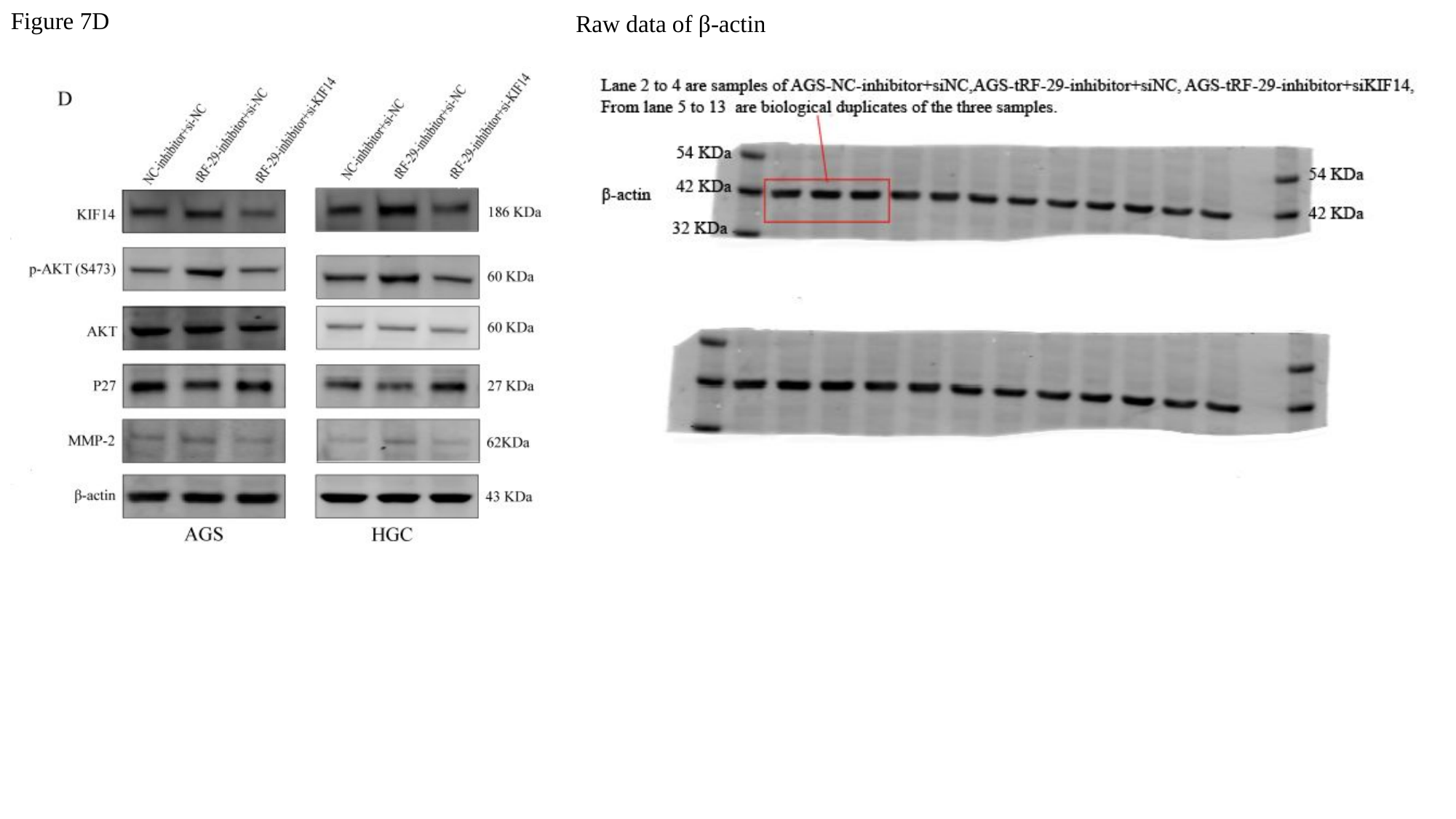

Figure 7D
Raw data of β-actin

## Slide 19
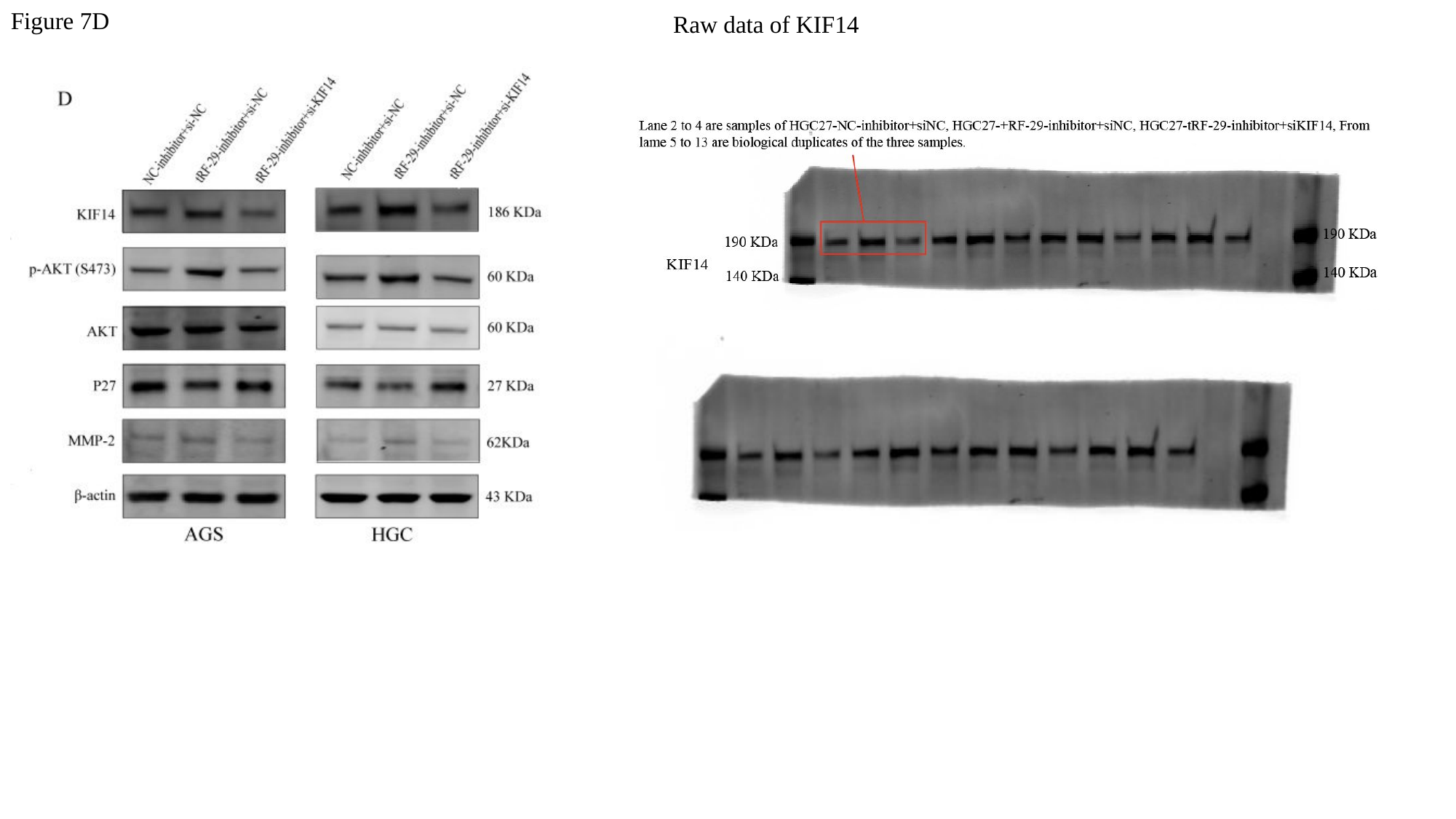

Figure 7D
Raw data of KIF14

## Slide 20
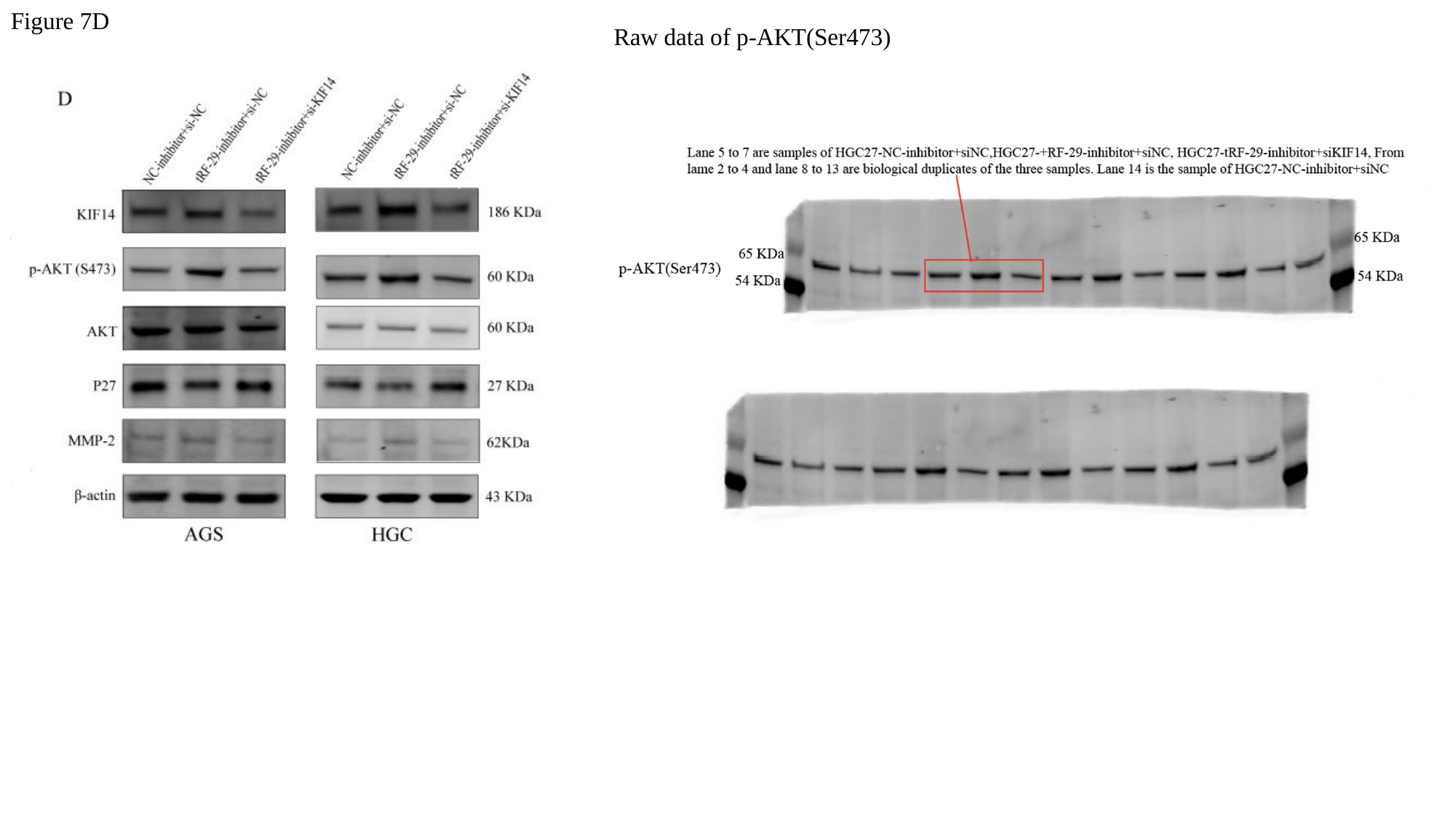

Figure 7D
Raw data of p-AKT(Ser473)

## Slide 21
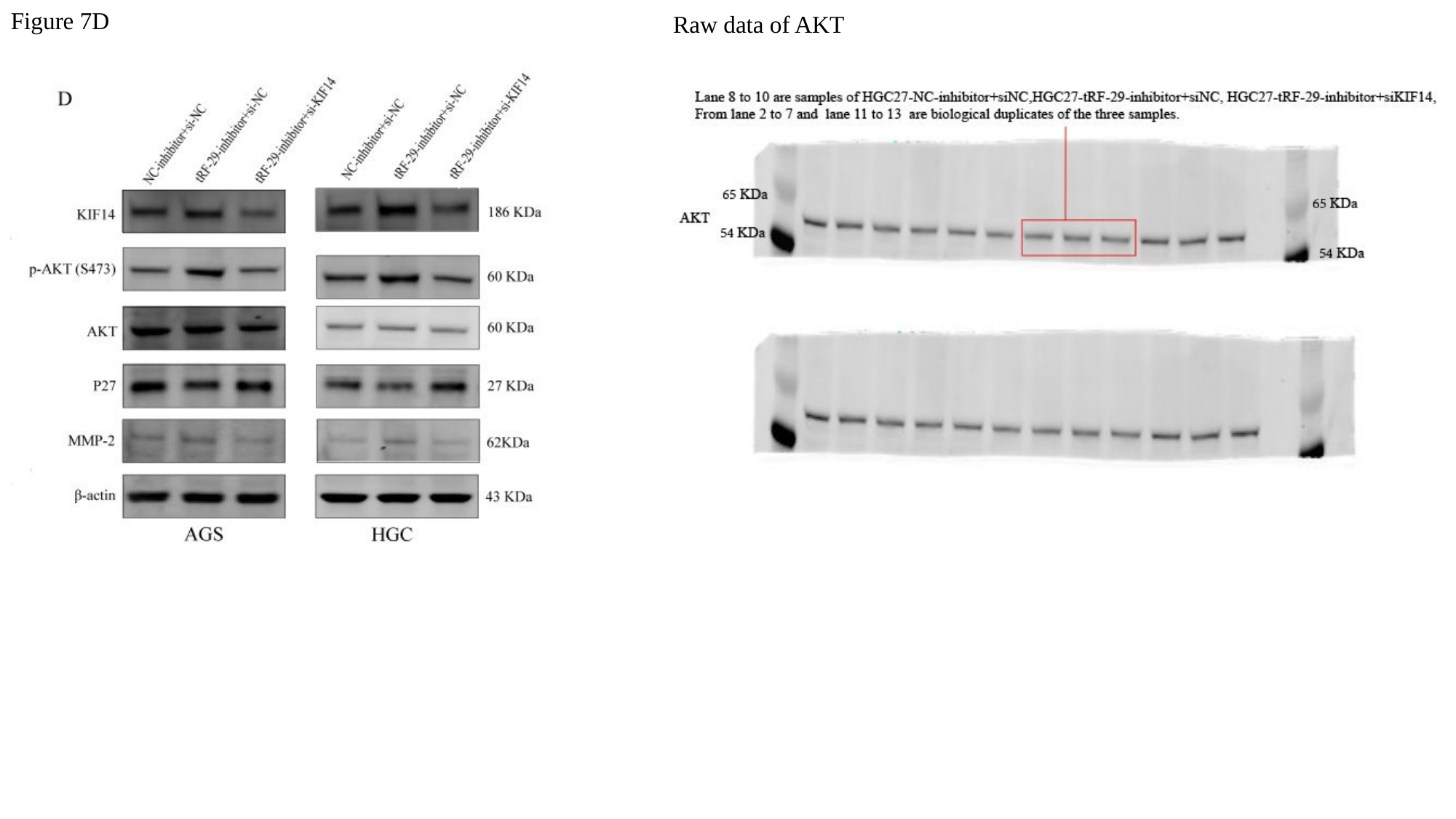

Figure 7D
Raw data of AKT

## Slide 22
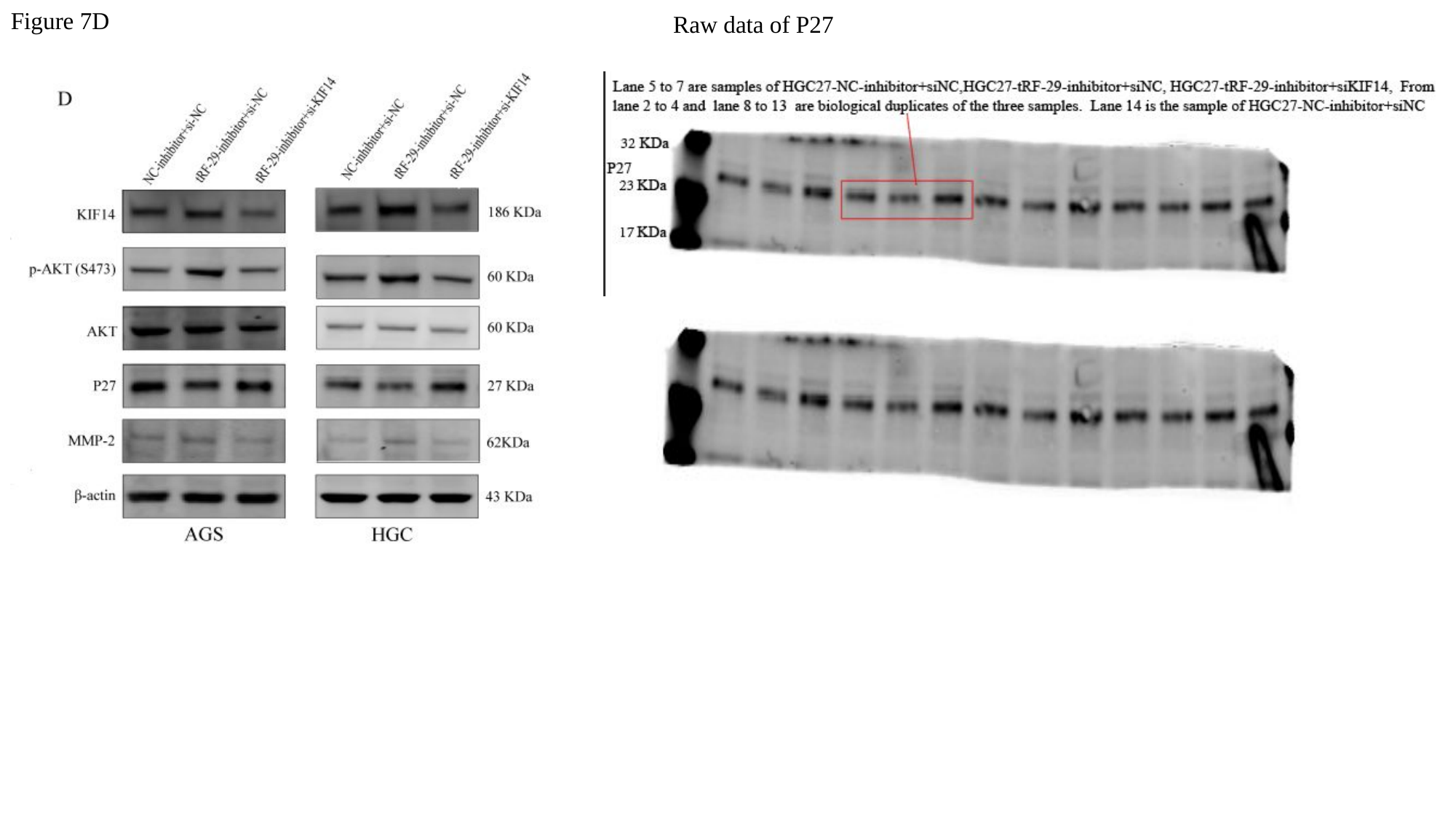

Figure 7D
Raw data of P27

## Slide 23
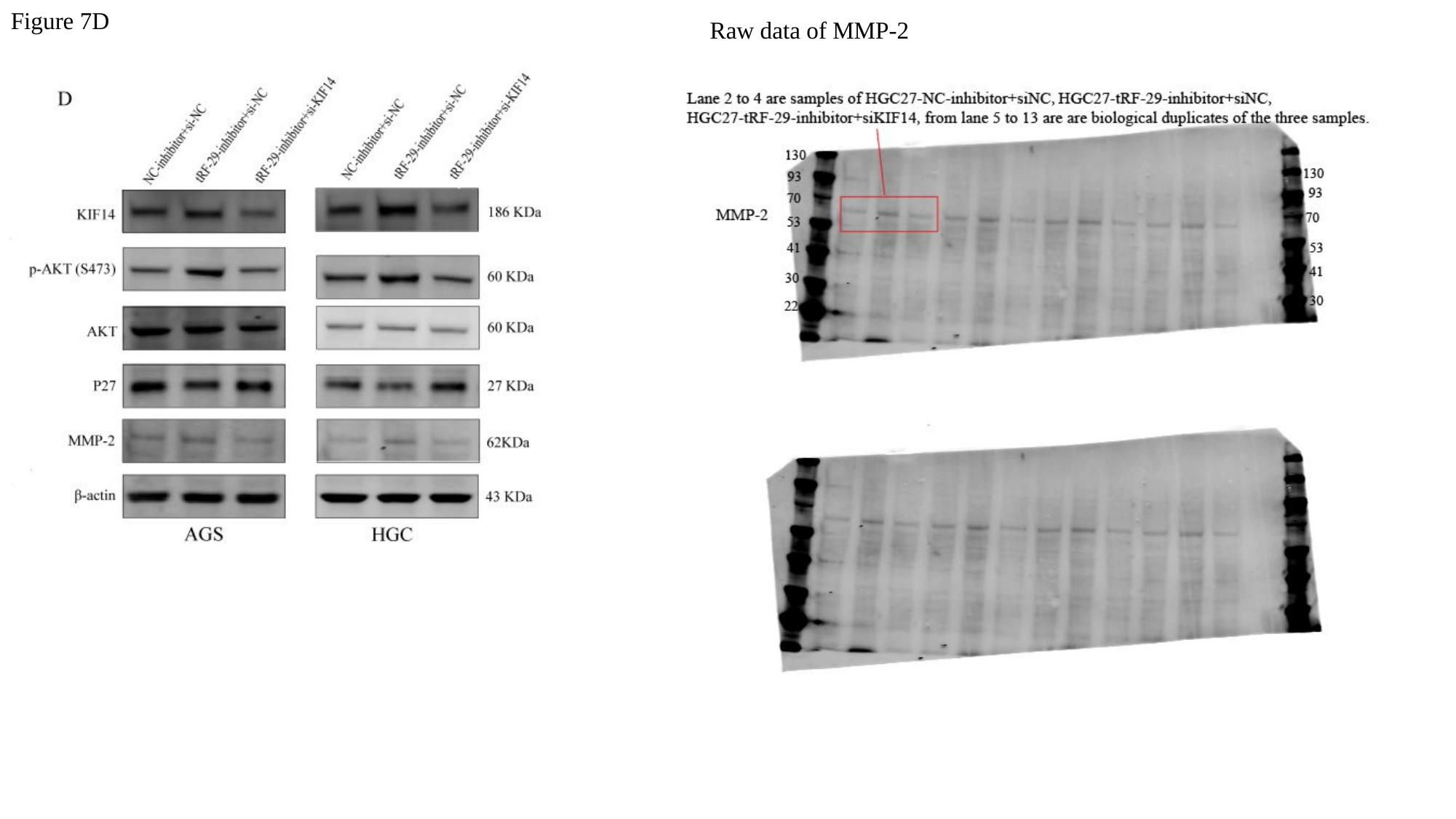

Figure 7D
Raw data of MMP-2

## Slide 24
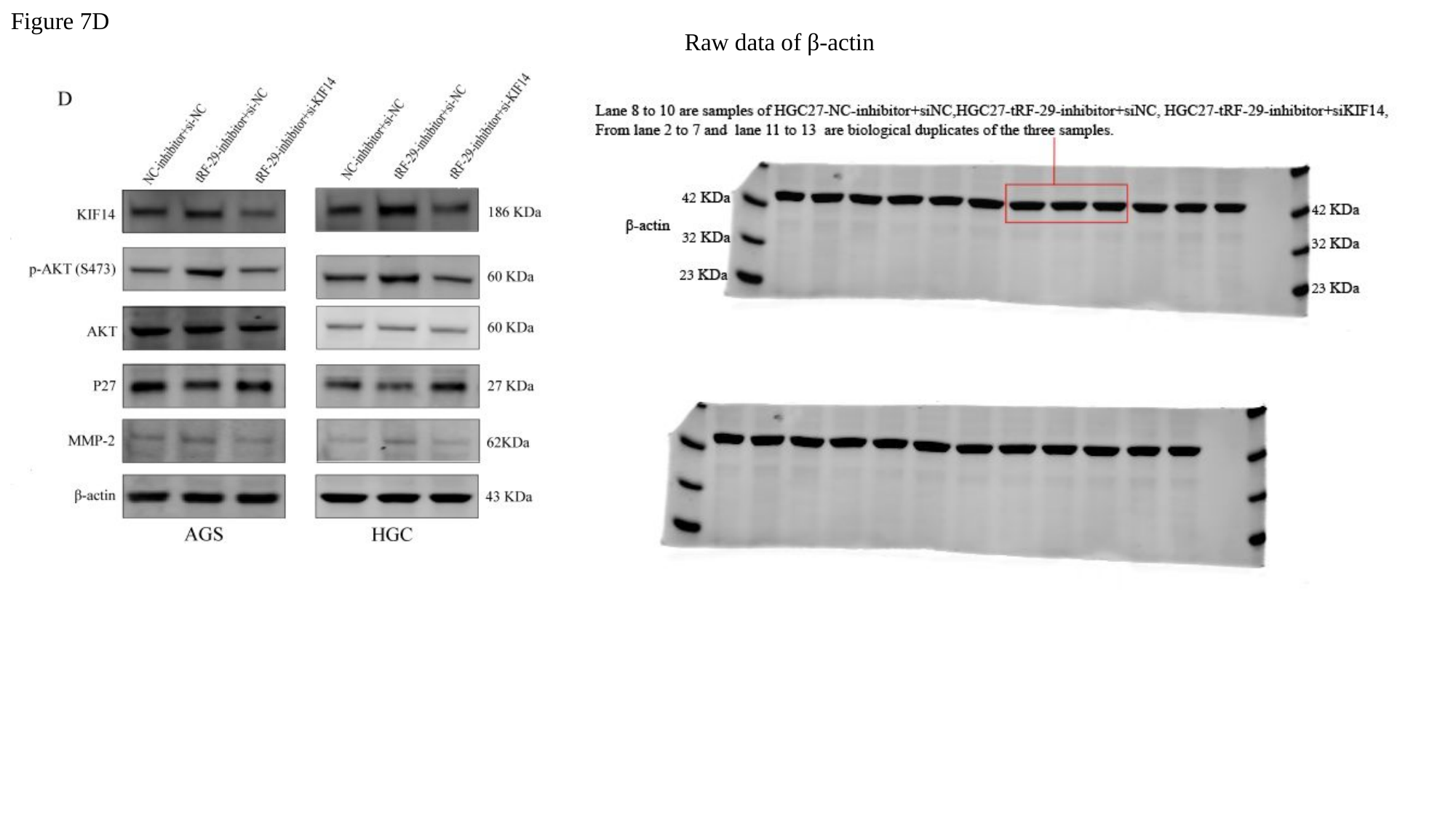

Figure 7D
Raw data of β-actin

## Slide 25
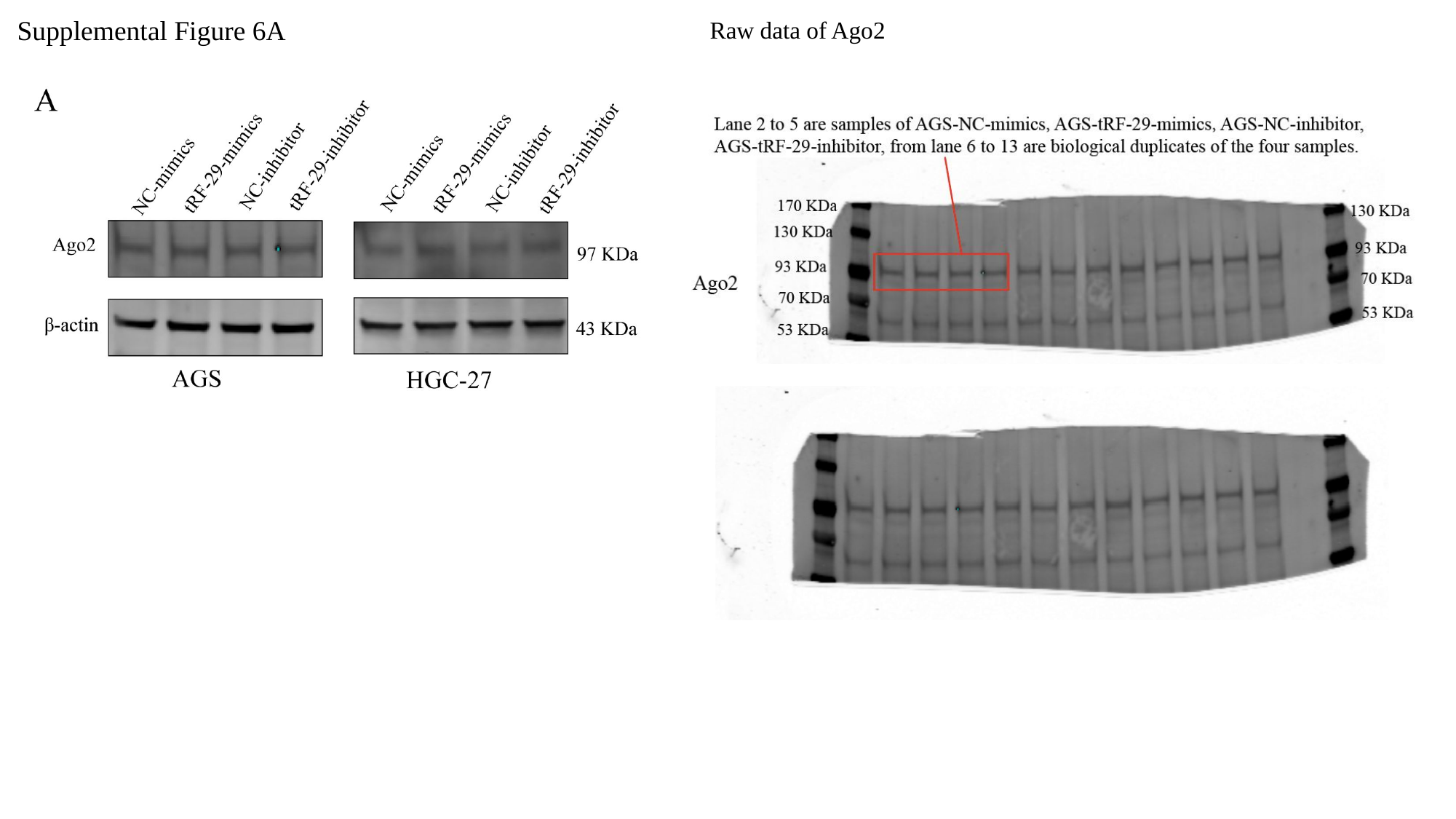

Supplemental Figure 6A
Raw data of Ago2

## Slide 26
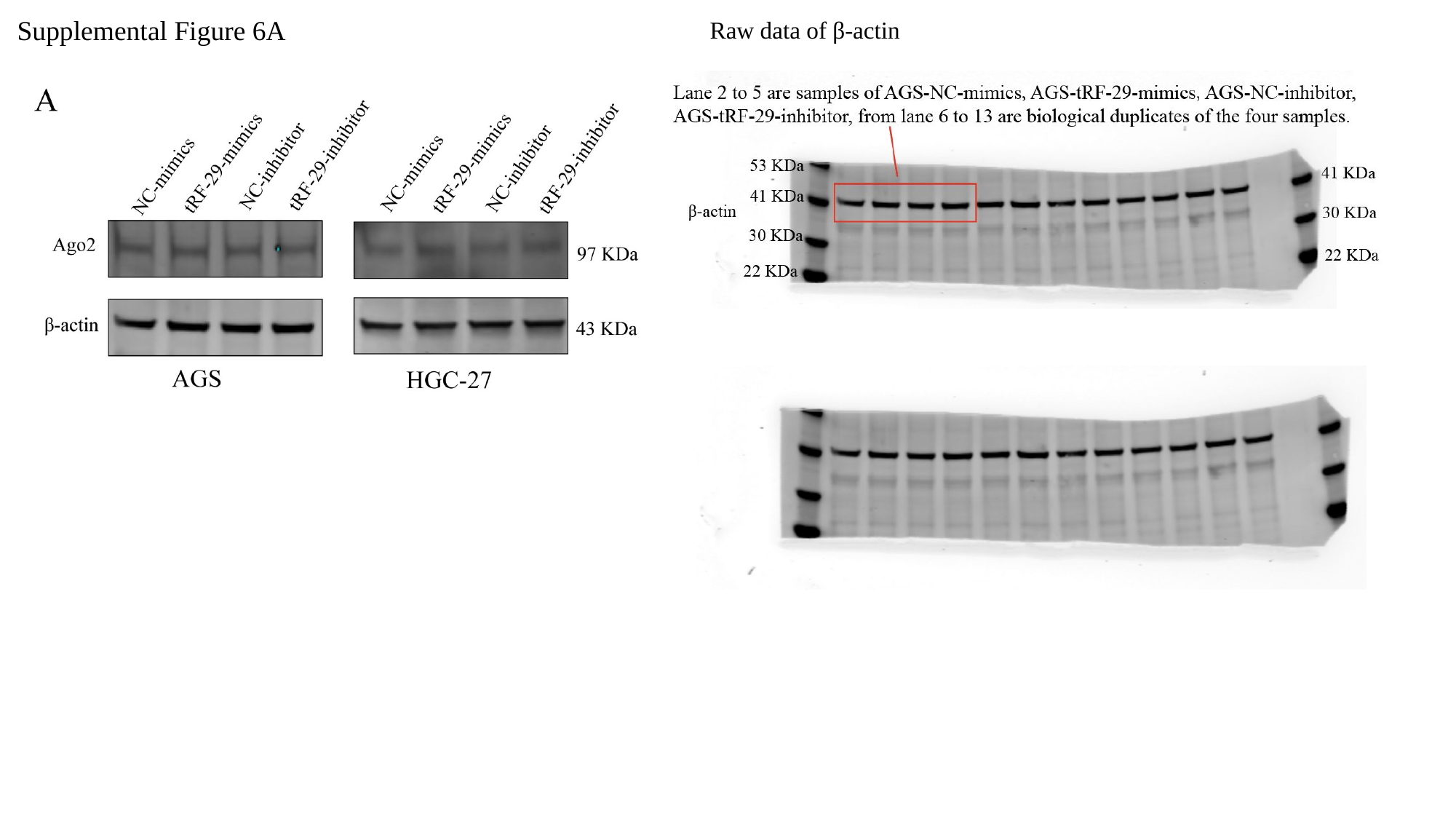

Supplemental Figure 6A
Raw data of β-actin

## Slide 27
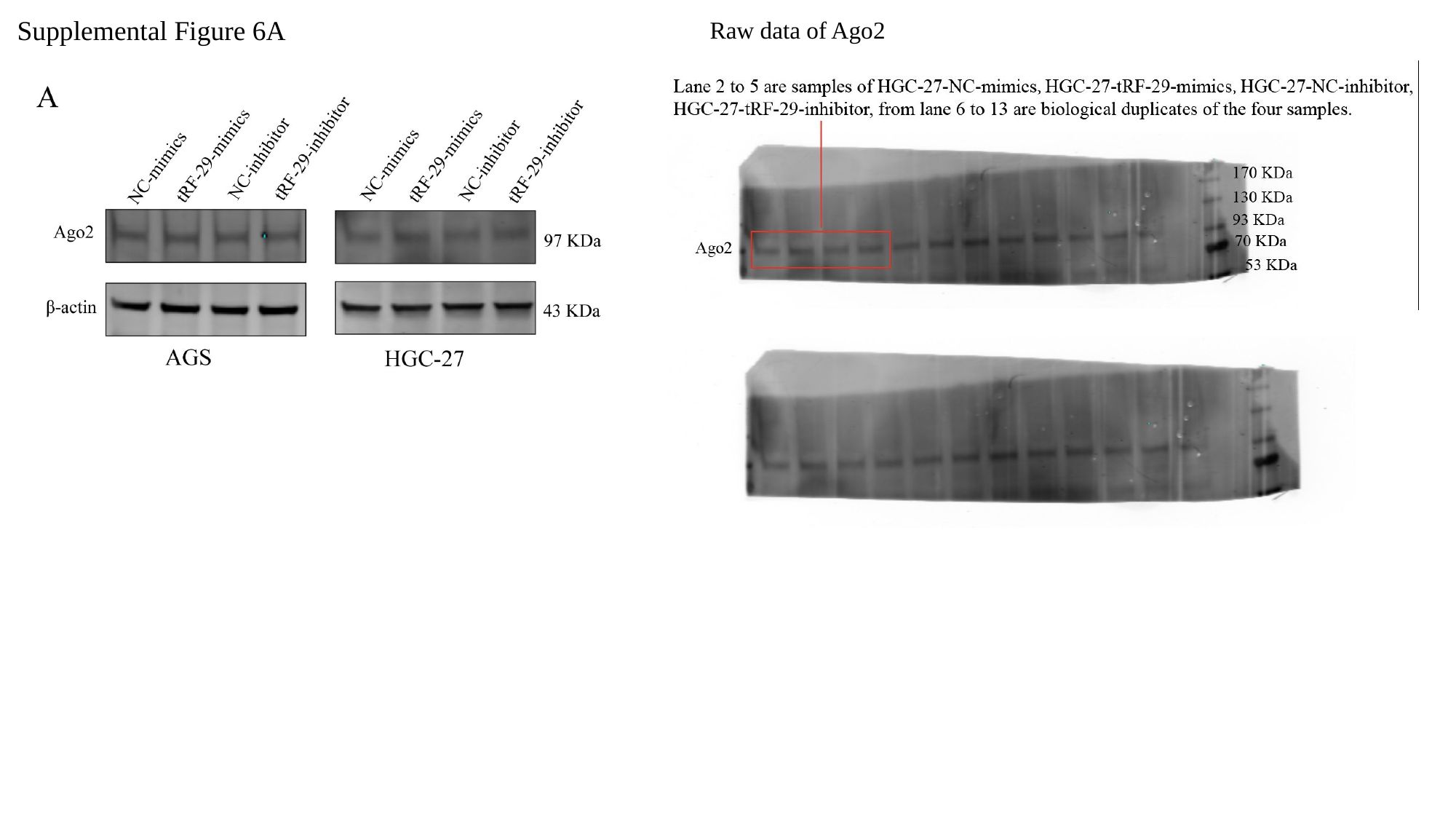

Supplemental Figure 6A
Raw data of Ago2

## Slide 28
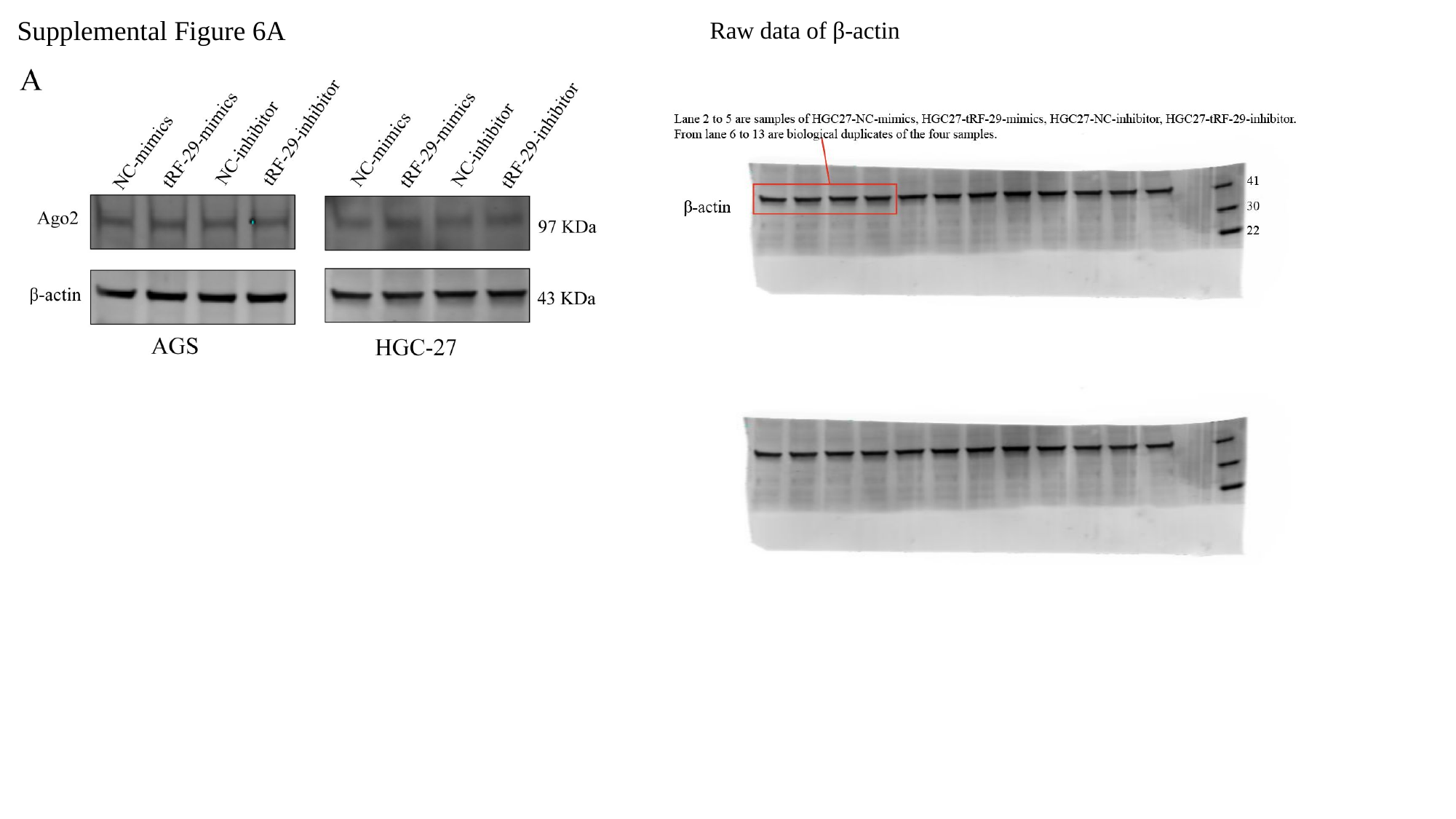

Supplemental Figure 6A
Raw data of β-actin
